# iACP: a sequence-based tool for identifying anticancer peptides

**DOI:** 10.18632/oncotarget.7815

**Published:** 2016-03-01

**Authors:** Wei Chen, Hui Ding, Pengmian Feng, Hao Lin, Kuo-Chen Chou

**Affiliations:** ^1^ Department of Physics, School of Sciences, Center for Genomics and Computational Biology, North China University of Science and Technology, Tangshan, China; ^2^ Key Laboratory for Neuro-Information of Ministry of Education, Center of Bioinformatics, School of Life Science and Technology, University of Electronic Science and Technology of China, Chengdu, China; ^3^ School of Public Health, North China University of Science and Technology, Tangshan, China; ^4^ Gordon Life Science Institute, Belmont, Massachusetts, United States of America; ^5^ Center of Excellence in Genomic Medicine Research (CEGMR), King Abdulaziz University, Jeddah, Saudi Arabia

**Keywords:** anticancer peptides, PseAAC, g-gap dipeptide mode, incremental feature selection, iACP webserver

## Abstract

Cancer remains a major killer worldwide. Traditional methods of cancer treatment are expensive and have some deleterious side effects on normal cells. Fortunately, the discovery of anticancer peptides (ACPs) has paved a new way for cancer treatment. With the explosive growth of peptide sequences generated in the post genomic age, it is highly desired to develop computational methods for rapidly and effectively identifying ACPs, so as to speed up their application in treating cancer. Here we report a sequence-based predictor called iACP developed by the approach of optimizing the g-gap dipeptide components. It was demonstrated by rigorous cross-validations that the new predictor remarkably outperformed the existing predictors for the same purpose in both overall accuracy and stability. For the convenience of most experimental scientists, a publicly accessible web-server for iACP has been established at http://lin.uestc.edu.cn/server/iACP, by which users can easily obtain their desired results.

## INTRODUCTION

Millions of people have been killed by cancer globally every year [1]. Although cancer can be treated with the combination of radiation therapy, targeted therapy and chemotherapy, these physical or chemical methods are expensive and have some deleterious side effects on normal cells [2, 3]. It has also been demonstrated that cancer cells begin to exhibit resistance towards current anticancer drugs [4]. Therefore, it is urgent to develop novel anticancer agents.

Because anticancer peptides (ACPs) do not impair the normal body physiological functions, they open promising perspective for the cancer treatment [5, 6]. The discovery of ACPs has provided an alternative approach to treat cancer. Despite some potential drawbacks during their development process, such as low *in vivo* stability and high costs for production [5], ACPs have some unique and exceptional advantages. This is because ACPs are naturally occurring biologics, and hence are safer than synthetic drugs, as well as have a greater efficacy, selectivity and specificity. In addition to the advantage of peptide drugs having no toxicity *in-vivo* under the normal physiological condition [7–9], ACPs are small peptides and usually contain 5 to 30 amino acids. Also, since ACPs are cationic in nature [10], they can interact with the anionic cell membrane components of cancer cells and then selectively kill cancer cells [10, 11]. Over the last decade, many peptide-based strategies against various tumor types have been pre-clinically used [12, 13], indicating that ACPs may become promising candidates for cancer treatments. In view of the fact that the clinical trials of ACPs are still under development, studies on ACPs action mechanisms are crucial for cancer treatment. Therefore, it is important for both basic research and drug development to discriminate ACPs from natural and artificially designed peptides.

Unfortunately, experimental identification and development of novel ACPs is extremely cost-ineffective and time-consuming. Besides, only few of them have been successfully translated into clinics [14]. Therefore, it is necessary to resort to computational methods. Actually, using amino acid composition and binary profiles as the input of support vector machine (SVM), Tyagi et al. [15] proposed a model to identify ACPs. Shortly afterwards, Hajisharifi et al. [16], using Chou's pseudo amino acid composition and the local alignment kernel based method, also proposed a model to do the same. Both methods yielded quite encouraging results and have indeed played an important role in stimulating the development of this area.

In considering the importance of ACPs to human beings’ health, the present study was initiated to further enhance the identification quality by proposing a new and more powerful predictor for the same purpose. Furthermore, to maximize the convenience for most experimental scientists, we have provided a user-friendly web-server and a step-by-step guide by which users can easily obtain their desired results without the need to go through the mathematical equations, which, however, are useful for those who want to use the current mathematical approach to develop other predictors in computational biology.

As demonstrated in a series of recent publications [17–29], to establish a really useful sequence-based statistical predictor for a biological system and also to make the presentation logically more clear and easier to follow, according to Chou's 5-step guidelines [30] we should make the following five procedures crystal clear: (1) benchmark dataset; (2) sample representation; (3) operation engine; (4) cross validation; (5) web-server. Below, let us elaborate how to deal with the five steps one-by one. To match the Journal's style, however, they are not exactly following the above order.

## RESULTS AND DISCUSSION

A new and more powerful sequence-based method, called iACP, was developed for predicting anti-cancer peptides.

### Comparison with other existing methods

The jackknife success rates achieved by iACP on the benchmark dataset (see Supporting Information S1) are given in Table 1, where for facilitating comparison, the rates reported by Hajisharifi et al. [16] are also listed. As we can see from Table 1, iACP outperformed the method by Hajisharifi et al.'s method in both Acc and MCC, indicating that the current predictor is not only able to achieve higher overall success rate, but also more stable.

**Table 1:** A comparison of the current method iACP with hajisharifi et al.'s method [16] on the same benchmark dataset (Online Supporting Information S1)

Prediction method	Validation method	Sn^c^ (%)	Sp^c^ (%)	Acc^c^ (%)	MCC^c^
iACP^a^	Jackknife test	89.86	98.54	95.06	0.897
	5-fold cross-validation	88.40	99.02	94.77	0.893
Hajisharifi et al.^b^	5-fold cross-validation	89.70	85.18	92.68	0.784

a Proposed in this paper.

b See ref. [16].

c See the section of “A set of four metrics”.

It should be noted that the rates reported by Hajisharifi et al. [16] were obtained by the 5-fold cross-validation rather than the rigorous jackknife rest and hence would lack objectiveness [30]. For the current case, the benchmark dataset contains 138 ACPs and 206 non-ACPs. According to the Eqs.28 and 29 in the review article [30], the number of possible combinations for conducting the 5-cross-validation would be more than 10^74^. Therefore, the rates reported by Hajisharifi et al. [16] were derived from an extremely small fraction of the possible combinations, and hence could not avoid arbitrariness. If the iACP predictor was also tested by the 5-fold cross-validation on the same benchmark dataset, however, we obtained Acc = 94.77% and MCC = 0.893, which are also remarkably higher than the corresponding rates by Hajisharifi et al.

To further verify the power of the current predictor, a comparison was also made between iACP and Tyagi et al.'s method AntiCP [15] on a same independent dataset (see Eq.2 and Supporting Information S2). As mentioned in the “Benchmark Dataset” section, none of the independent data occurs in the dataset used to train the current predictor iACP. Accordingly, there is no memory advantage [31] whatsoever to iACP. The results thus obtained are given in Table 2, from which we can observe the following. The overall accuracy Acc and Matthews correlation coefficient MCC obtained by iACP are 92.67% and 0.88, respectively. They are remarkably higher than the corresponding rates obtained by the AntiCP method [15], which are 50.00% and 0.00 for its module 1 and 66.33% and 0.36 for its module 2, respectively. The detailed predictive results thus obtained are given in Supporting Information S3. The above results indicate that the proposed predictor iACP is indeed quite promising or at least can play a complimentary role to the existing state-of-the art methods in this area [15, 16].

**Table 2:** A comparison of the current method with the one by Tyagi et al. [15] on the same independent dataset given in Supporting Information S2, which contains 150 anticancer peptides and 150 non-anticancer peptides, and none of the peptides there occurs in the Supporting Information S1 used to train iACP

Prediction method		Sn^c^ (%)	Sp^c^ (%)	Acc^c^ (%)	MCC^c^
iACP ^a^		93.33	92.00	92.67	0.85
Tyagi et al.^b^	Module 1	100	0	50	0
	Module 2	89.33	45.33	66.33	0.36

a Proposed in this paper.

b Available at http://crdd.osdd.net/raghava/anticp/multi_pep.php.

c See the footnote c of Table 1.

### A heat map analysis

**Figure 1:**
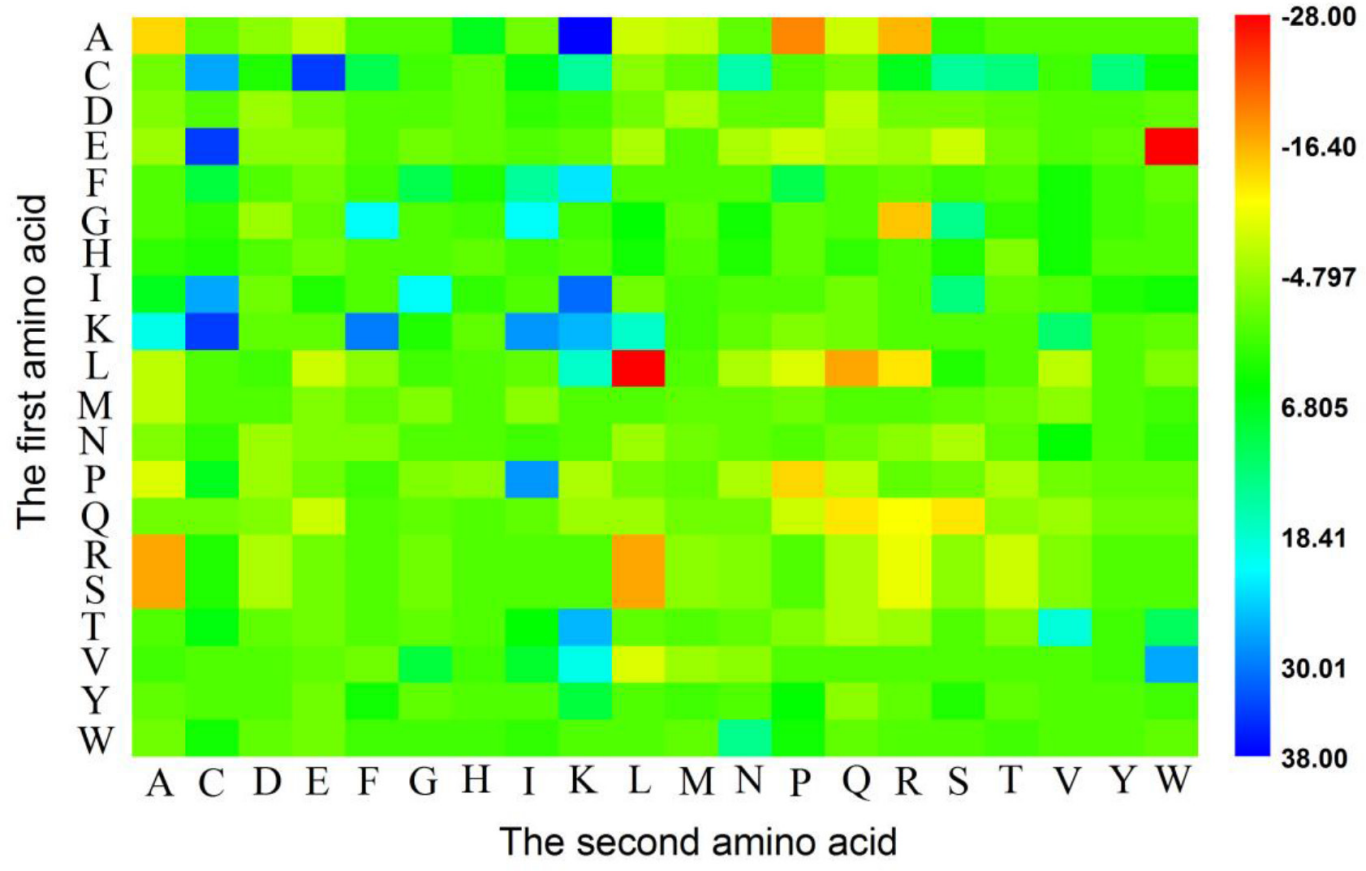
A heat map or chromaticity diagram for the *F* values of the 400 1-gap dipeptides The blue boxes indicate that the features are enriched in anticancer peptide, while the red boxes indicate that the features are enriched in non-anticancer peptide. See the text for more explanation.

Why could the current model achieve so high success rates? To address this problem, let us perform an intuitive graphical analysis. Using graphical approaches to study biological problems can provide very useful insights for in-depth analyzing complicated relations in these systems, as demonstrated by a series of previous studies on various important biological topics, including enzyme-catalyzed reactions [32–38], protein folding kinetics and folding rates [39–42], inhibition of HIV-1 reverse transcriptase [43–46], inhibition kinetics of processive nucleic acid polymerases and nucleases [47], derivation of steady-state reaction system [48], studying drug metabolism systems [49], analyzing codon usage [50–52], base frequencies in the anti-sense strands [53], and protein sequence evolution [54], as well as using wenxiang graphs [55] to analyze protein-protein interactions [56, 57]. In this study, the heat map [58] was used to conduct the analysis as given in Figure 1, where the row and column of the heat map represent the first and second amino acid residues of the 1-gap dipeptides, respectively. Each element in the heat map represents one of the 400 1-gap dipeptides and is colorized according to its *F*-score (cf. the “Feature Selection” section later). The features in blue boxes are positively correlated with ACP, while those in red boxes are positively correlated with non-ACP. It was observed that the absolute values of *F*-scores for most of the 1-gap dipeptides are near 0 (in green box), indicating that these features are irrelevant to the anticancer peptide predictions. While the residues Cys (C), Glu (E), Phe (F), Gly (G), Ile (I) and Lys (K) are abundant in ACP compared to non-ACP.

Different from normal cell membranes, cancer cell membranes carry a net negative charge [59, 60]. It has been demonstrated that the membrane interaction and insertion of membrane-active peptides could be due to their conformation [61], which can be associated to a particular order of amino acids. In other words, the 1-gap dipeptides compositions may be associated with the anticancer properties of ACPs, and hence may also be used to account for the ability of killing cancer cells.

### Web-server guide

For the convenience of most experimental scientists, a publicly accessible web-server for iACP has been established. Furthermore, to maximize the user's convenience, a step-by-step guide on how to use the web-server is given bellow.

**Figure 2:**
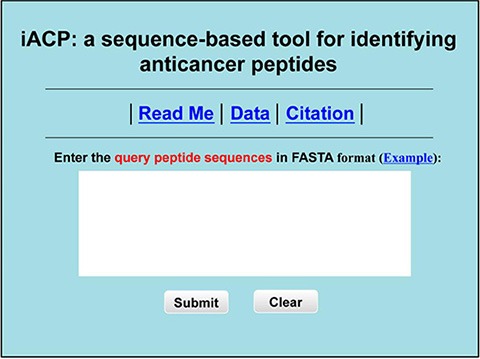
A semi-screenshot to show the top page of the iACP web-server Its website address is at http://lin.uestc.edu.cn/server/iACP.

Step 1. Open the web server at http://lin.uestc.edu.cn/server/iACP and you will see the top page of iACP on your computer screen, as shown in Figure 2. Click on the Read Me button to see a brief introduction about the predictor and the caveat when using it.

Step 2. Either type or copy/paste the query peptide sequences into the input box at the center of Figure 2. The input sequence should be in the FASTA format. A sequence in FASTA format consists of a single initial line beginning with a greater-than symbol (“>”) in the first column, followed by lines of sequence data. The sequence ends if another line starting with a “>” appears; this indicates the start of another sequence. Example sequences in FASTA format can be seen by clicking on the Example button right above the input box.

Step 3. Click on the Submit button to see the predicted result. For example, if you use the query sequences in the Example window as the input, you will see the following shown on the screen: the outcome for the 1st query example is “Anticancer peptide”; the outcome for the 2nd query sample is “non-Anticancer peptide”. All these results are fully consistent with the experimental observations.

Step 4. Click on the Data button to download the benchmark dataset or independent dataset used in this study to train and test the iACP predictor.

Step 5. Click on the Citation button to find the relevant papers documenting the detailed development and algorithm of iACP.

## MATERIALS AND METHODS

### Benchmark dataset

The benchmark dataset S used in this study can be formulated as

$$S=S^{+}\cup S^{-}$$  (1)

where the positive and negative subsets, $S^{+}$ and $S^{-}$, contain respectively anticancer and non-anticancer peptides, while the symbol ∪ represents the union in the set theory. As elucidated by a comprehensive review [31], there is no need to separate the benchmark dataset into a training dataset and a testing dataset if the predictor to be developed will be tested by the jackknife test or subsampling (K-fold) cross-validation test since the outcome thus obtained is actually from a combination of many different independent dataset tests. In order to have a high quality benchmark dataset, the samples in the positive subset were taken from Hajisharifi et al. [16] that contain 138 anticancer peptides, which had been derived from the antimicrobial peptide database [62] as well as the existing literatures. The samples in the negative subset, however, were constructed as follows. In view of the fact that the peptides with anticancer activity are generally secretory [63], the non-anticancer peptides can be selected from the non-secretory proteins deposited in Universal Protein Resource. To avoid redundancy and reduce the homology bias, peptides with more than 90% sequence similarity were removed by using the CD-HIT program [64]. After such a screening procedure, we finally obtained 206 non-anticancer peptides for the negative subset. The 138 anticancer peptides and 206 non-anticancer peptides are given in Supporting Information S1.

**Figure 3:**
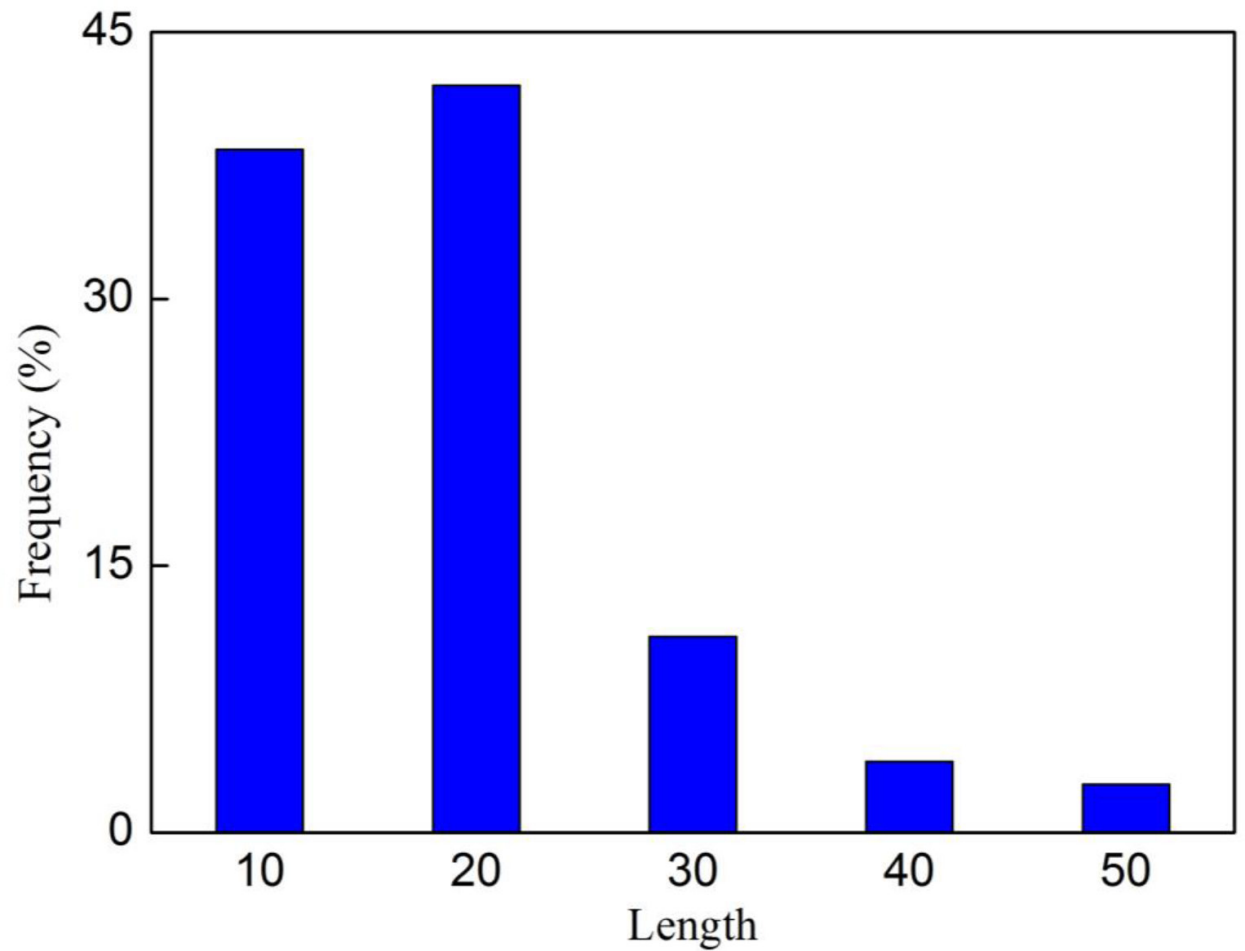
The length distribution of the 138 anticancer peptides in Supporting Information S1

The statistical distribution of the length for the 138 anticancer peptides is given in Figure 3, from which we can see that most (∼80%) of them are with the length less than 30 amino acids.

As clearly pointed out in the beginning of this section, the independent dataset is not absolutely needed for validating a predictor via the jackknife or K-fold cross-validation, but as a demonstration to show how to use the proposed predictor, it may be of help [65] to also construct an independent dataset $S_{\text{Ind}}$ as formulated by

$$S_{\text{Ind}}=S_{\text{Ind}}^{+}\cup S_{\text{Ind}}^{-}$$  (2)

where the samples in $S_{\text{Ind}}^{+}$ and $S_{\text{Ind}}^{-}$ were fetched from the dataset used by Tyagi et al. [15] and the recent CancerPPD database [66] according to the following criteria: (1) none of the anticancer peptides in $S_{\text{Ind}}^{+}$ occurs in $S^{+}$; (2) none of the non-anticancer peptides in $S_{\text{Ind}}^{-}$ occurs in $S^{-}$; (3) neither the included peptides in $S_{\text{Ind}}$ contains illegal single-letter amino acid codes such as “B”, “U”, “X”, and “Z”, nor the peptides in $S_{\text{Ind}}$ has ≥ 90% pairwise sequence identity to any other in the benchmark dataset of Eq.1. By strictly following the aforementioned procedures, we finally obtained an independent dataset $S_{\text{Ind}}$, in which the positive subset $S_{\text{Ind}}^{+}$ contains 150 anticancer peptides, and the negative subset $S_{\text{Ind}}^{-}$ contains 150 non-anticancer peptides. See Supporting Information S2 for the detailed information. Actually, all the datasets used in this study can also be directly downloaded from the website at http://lin.uestc.edu.cn/server/iACP/data.

### Pseudo amino acid composition with G-Gap dipeptide mode

Given a peptide, how can we translate it into a mathematical expression for statistical analysis? Obviously, the most straightforward way to formulate a peptide sample P with *L* residues is to use the sequential model as typically given by

$$P=R_{1}R_{2}R_{3}R_{4}\cdots R_{L-1}R_{L}$$  (3)

where R_1_ represents the 1st residue in the peptide, R_2_ the 2nd residue, and so forth. With the sequential model to represent a peptide, not only all its constituent amino acids but also their sequence order or pattern can be precisely defined. Various existing sequence-similarity-search-based tools such as BLAST [67, 68] can be utilized to identify whether a query peptide belongs to anticancer or not. Although quite straightforward and simple, this kind of intuitive approach failed to work when a query peptide sample did not have significant sequence similarity to any of the character-known peptides [31].

To cope with this problem, investigators could not help but resort to the discrete or vector model. Another reason for them to shift their efforts from the intuitive sequential model to various vector models is that statistical samples formulated based on a vector model can be directly handled by all the existing machine-learning algorithms, such as the optimization approach [69], correlation coefficient method [70], correlation angle approach [71], neural network [72], covariance discriminant (CD) [73, 74], SLLE algorithm [75], nearest neighbor (NN) [76]; OET-KNN [77], K-nearest neighbor (KNN) [78, 79]; random forest [80], fuzzy K-nearest neighbor [78], conditional random field [81], ML-KNN algorithm [82], and support vector machine (SVM) [83].

The simplest vector used to represent a peptide sample is its amino acid composition (AAC), as given below

$$R={\left[f_{1}\;f_{2}\;\cdots \;f_{20}\right]}^{T}$$  (4)

where *f_i_* (i = 1, 2, …, 20) is the normalized occurrence frequency of the *i*-th type of native amino acid in the peptide chain, and T the transpose operator. The AAC model was used by many in predicting various attributes of proteins (see, e.g., [69, 84–87]). As we can see from Eq.4, however, if using AAC to represent a peptide sample, all its sequence order information would be completely lost, and hence the prediction quality will be substantially limited.

How can we formulate a peptide with a vector that can effectively reflect its sequence pattern information or capture its key features closely correlated with the predicted target? One of the feasible ways to address such a dilemma is to adopt the approach of pseudo amino acid composition [88, 89] or Chou's PseAAC [90–92]. Ever since the concept of PseAAC was proposed in 2001 [88], it has been penetrating into nearly all the fields of protein attribute predictions (see, e.g., [93–107]) and a long list of papers cited in the References section of [92, 108] as well as a recent review [109]). It has also been used in some disciplines of drug development and biomedicine [110] as well as drug-target area [111, 112].

According to [30], the general PseAAC is formulated by

$$P={\left[\Psi_{1}\;\Psi_{2}\;\cdots \;\Psi_{u}\;\cdots \;\Psi_{\Omega }\right]}^{T}\;$$  (5)

where the component Ψ_*u*_(*u* = 1, 2,⋯, Ω) and the dimension Ù will depend on how to extract the features from the peptide sequences concerned. In the current study, we are to use the following approach to define the components in Eq.5.

The proximate dipeptide composition has been widely used in computational proteomics [113–115]. However, the intrinsic properties of protein sequences are usually reflected by the higher tier correlation [88] of the constituent residues due to the long-range interaction. Accordingly, instead of the proximate dipeptide composition, we consider the *g*-gap dipeptide composition, which has been demonstrated quite promising for identifying protein attributes [116, 117].

For the peptide P as defined in Eq.3, its *g*-gap dipeptide composition can be generally expressed as

$$\begin{array}{l} P={\left[d_{1}^{g}\;d_{2}^{g}\;\cdots \;d_{u}^{g}\;\cdots \;d_{u}^{400}\right]}^{T}\\ =\{\begin{array}{cc} \text{proximate dipeptide composition} & \text{when }g=0\;\\ \text{one}-\text{gap dipeptide composition}, & \text{when }g=1\\ \text{two}-\text{gap dipeptide composition}, & \text{when }g=2\\ \text{three}-\text{gap dipeptide composition}, & \text{when }g=3\\ \text{four}-\text{gap dipeptide composition}, & \text{when }g=4\\ \vdots & \vdots \end{array} \end{array}$$  (6)

where $d_{u}^{g}$ denotes the occurrence frequency of the *u*-th *g*-gap dipeptide in the peptide as given by

$$d_{u}^{g}=\frac{n_{u}^{g}}{\sum_{u=1}^{400}n_{u}^{g}}=\frac{n_{u}^{g}}{\left(L-g-1\right)}\;(g=0,1,2,3,4,\cdots )$$  (7)

where $n_{u}^{g}$ denotes the number of the *u*-th *g*-gap dipeptide. Since the sequences of anticancer peptides are not long (see Figure 3), the range for *g* we need to consider in the current study is up to 4: the case for *g* = 0 is none but the dipeptide composition formed by the nearest residues as considered in [118, 119]; *g =* 1 that formed by the 2nd nearest residues as considered in [120]; *g* = 2 that formed by the 3rd nearest residues; and so forth. Thus, each of the components in the general PseAAC of Eq.5 can be uniquely defined as

$$\{\begin{array}{c} \Psi_{u}=d_{u}^{g}\\ \Omega =400 \end{array}\;(u=1,2,\cdots ,\Omega ;\;g=0,1,2,3,\text{or }4)$$  (8)

### SVM (support vector machine) classifier

The SVM classification algorithm has been widely used in the realm of bioinformatics (see, e.g., [21, 24, 28, 29, 83, 121–124]). The basic idea of SVM is to construct a separating hyper-plane to maximize the margin between the positive dataset and negative dataset. For a brief formulation of SVM and how it works, see the papers [125, 126]; for more details about SVM, see a monograph [127].

The software of SVM used in the current study was downloaded from the LIBSVM 2.84 package [128] at http://www.csie.ntu.edu.tw/~cjlin/libsvm. Because of its effectiveness and speed in nonlinear classification process, the radial basis kernel function (RBF) was selected to perform the prediction. In the SVM operation engine, the regularization parameter *C* and the kernel width parameter *γ* can be determined via an optimization procedure using the grid search approach. In this study, their optimal values were found to be *C* = 2 and *γ* = 0.125, respectively.

### Performance evaluation

The following two things are important for evaluating the quality of a statistical predictor: (1) what kind of cross-validation method should be adopted to test it; (2) what kind of metrics should be used to measure its accuracy.

### Jackknife cross-validation

Three cross-validation test methods are often adopted in literature to test a statistical predictor: independent dataset test, sub-sampling (or K-fold cross-validation) test, and jackknife test [129]. Among the three, however, the jackknife test is deemed the least arbitrary and most objective because it can always yield a unique outcome for a given benchmark dataset as demonstrated by the equations 28–32 in a review paper [30]. Accordingly, the jackknife test has been increasingly used and widely recognized by investigators to examine various predictors (see, e.g., [96, 103, 104, 130–134]). In view of this, the jackknife test was also adopted here to examine the proposed model.

### A set of four metrics

To provide a more intuitive and easier-to-understand method to measure the prediction quality, the following set of four metrics based on the formulation used by Chou [135] in studying signal peptide prediction was adopted. According to Chou's formulation, the sensitivity, specificity, overall accuracy, and Matthews correlation coefficient can be expressed as [17, 23, 122, 136–139].

$$\{\begin{array}{cc} \text{Sn}=1-\frac{N_{-}^{+}}{N^{+}} & 0\leq \text{Sn}\leq 1\\ \text{Sp}=1-\frac{N_{+}^{-}}{N^{-}} & 0\leq \text{Sp}\leq 1\\ \text{Acc}=\;\Lambda \;=1-\frac{N_{-}^{+}+N_{+}^{-}}{N^{+}+N^{-}} & 0\leq \text{Acc}\leq 1\\ \text{MCC}=\;\frac{1-(\frac{N_{-}^{+}}{N^{+}}+\frac{N_{+}^{-}}{N^{-}})}{\sqrt{\left(1+\frac{N_{+}^{-}-N_{-}^{+}}{N^{+}})(1+\frac{N_{-}^{+}-N_{+}^{-}}{N^{-}}\right)}} & -1\leq \text{MCC}\leq 1 \end{array}$$  (9)

where *N*^+^ is the total number of the anticancer peptides investigated while $N_{-}^{+}$ the number of anticancer peptides incorrectly predicted as the non-anticancer peptides; *N*^−^ the total number of the non-anticancer peptides investigated while $N_{-}^{+}$ the number of the non-anticancer peptides incorrectly predicted as the anticancer peptides. According to Eq.9 we can easily see the following. When $N_{-}^{+}=0$ meaning none of the anticancer peptides was mispredicted to be a non-anticancer peptide, we have the sensitivity Sn = 1; while $N_{-}^{+}=N^{+}$ meaning that all the anticancer peptides were mispredicted to be the non-anticancer peptides, we have the sensitivity Sn = 0. Likewise, when $N_{+}^{-}=0$ meaning none of the non-anticancer peptides was mispredicted, we have the specificity Sp = 1; while $N_{-}^{+}=N^{-}$ meaning all the non-anticancer peptides were incorrectly predicted as anticancer peptides, we have the specificity Sp = 0. When $N_{-}^{+}=N_{+}^{-}=0$ meaning that none of the anticancer peptides in the positive dataset $S^{+}$ and none of the non-anticancer peptides in the negative dataset $S^{-}$ was incorrectly predicted, we have the overall accuracy Acc = 1; while $N_{-}^{+}=N^{+}$ and $N_{-}^{+}=N^{-}$ meaning that all the anticancer peptides in the positive dataset and all the non-anticancer peptides in the negative dataset were mispredicted, we have the overall accuracy Acc = 0. The Matthews correlation coefficient MCC is usually used for measuring the quality of binary (two-class) classifications. When $N_{-}^{+}=N_{+}^{-}=0$ meaning that none of the anticancer peptides in the positive dataset and none of the non-anticancer peptides in the negative dataset was mispredicted, we have MCC = 1; when $N_{-}^{+}=N^{+}/2$ and $N_{+}^{-}=N^{-}/2$ we have MCC = 0 meaning no better than random prediction; when $N_{-}^{+}=N^{+}$ and $N_{+}^{-}=N^{-}$ we have MCC = −1 meaning total disagreement between prediction and observation. As we can see from the above discussion, it is much more intuitive and easier-to-understand when using Eq.9 to examine a predictor for its four metrics, particularly for its Mathew's correlation coefficient. Note that, of the four metrics in Eq.9, the most important are the Acc and MCC: the former reflects the overall accuracy of a predictor; while the latter, its stability in practical applications. The metrics Sn and Sp are used to measure a predictor from two different angles, and they are actually constrained with each other [140]. Accordingly, it is meaningless to use only one of the two for comparing the quality of two predictors. In other words, a meaningful comparison in this regard should count the rates of both Sn and Sp, or even better the rate of their combination that is none but MCC.

It should be pointed out, however, the set of equations defined in Eq.9 is valid only for the single-label systems. For the multi-label systems whose emergence has become more frequent in system biology [65, 141, 142] and system medicine [143], a completely different set of metrics is needed as elucidated in [82].

### Feature selection

Inclusion of redundant and noisy information would lead to poor predicted results. To improve the prediction quality, the ANOVA (analysis of variance) procedure was performed to select the optimal features among the *g*-gap dipeptide compositions (see Eq.6). ANOVA has been used for feature selection in computational proteomics [117]. The principle of ANOVA is to measure the feature variances by calculating the ratio (*F*-value) of features between groups and within groups [144]. The *F*-value of the ξ-th *g*-gap dipeptide in benchmark dataset is defined by

$$F\left(\xi \right)=S_{{}_{B}}^{{}^{2}}\left(\xi \right)/S_{{}_{W}}^{{}_{2}}\left(\xi \right)$$  (10)

where $S_{B}^{2}\left(\xi \right)$ and $S_{W}^{2}\left(\xi \right)$ denote the sample variance between groups (also called Means Square Between or MSB) and sample variance within groups (also called Mean Square Within or MSW), respectively. They can be calculated according to the following equations

$$S_{B}^{2}\left(\xi \right)=\frac{1}{df_{B}}\sum_{i=1}^{K}m_{i}{\left(\frac{\sum_{j=1}^{m_{i}}f_{\xi }^{g}(i,j)}{m_{i}}-\frac{\sum_{i=1}^{K}\sum_{j=1}^{m_{i}}f_{\xi }^{g}(i,j)}{\sum_{i=1}^{K}m_{i}}\right)}^{2}$$  (11)

and

$$S_{W}^{2}(\xi )=\frac{1}{df_{W}}\sum_{i=1}^{K}\sum_{j=1}^{m_{i}}{\left(f_{\xi }^{g}(i,j)-\frac{\sum_{i=1}^{K}\sum_{j=1}^{m_{i}}f_{\xi }^{g}(i,j)}{\sum_{i=1}^{K}m_{i}})\right)}^{2}$$  (12)

where $df_{B}=K-1$ and $df_{W}=M-K$ are degrees of freedom for MSB and MSW, respectively; *K* and *M* represent the number of groups (for the current case *K* = 2) and total number of samples (for the current case *M* = 344), respectively; $f_{\xi }^{g}(i,j)$ denotes the frequency of the ξ-*th g*-gap dipeptide of the *j*-th sample in the *i*-th group; *m_i_* denotes the number of samples in the *i*-th group (for the current case *m*_1_=138, *m*_2_= 206).

The value of *F*(ξ) in Eq.10 reveals the correlation between the ξ-*th* feature and the group variables: the larger the feature *F*(ξ) is, the more relevant it is to the target concerned. The features thus ranked according their values from high to low reflect the order of their importance.

Based on the aforementioned order, we used the Incremental Feature Selection (IFS) to determine the optimal number of features. The IFS approach has been used to predict protein domain [145] and antimicrobial peptides [146], as well as identify colorectal cancer related genes [147] and classify hepatocellular cirrhosis and carcinoma [148].

During the IFS procedure, features in the ranked feature set were added one by one from high to low. A new feature set was composed when one feature had been added. By adding these features sequentially according to their descending order, 400 feature sets will be obtained. The τ-th feature set can be formulated as

$$S_{\tau }=\{\begin{array}{cccc} f_{1} & f_{2} & \cdots & f_{\tau } \end{array}\}\;(1\leq \tau \leq 400)\;$$  (13)

For each of the 400 feature-sets, an SVM-based model was constructed and examined using the 5-fold cross-validation test on the benchmark dataset. By doing so, we can obtain an IFS curve in a 2D Cartesian coordinate system with index τ as its X-coordinate and the accuracy rate as its Y-coordinate. The optimal feature set is expressed as

$$S_{\Theta }=\left\{\begin{array}{cccc} f_{1} & f_{2} & \cdots & f_{\Theta } \end{array}\right\}$$  (14)

with which the IFS curve reaches its peak. And such a set of features will be used for further study.

### Optimal g-gap dipeptide set

**Figure 4:**
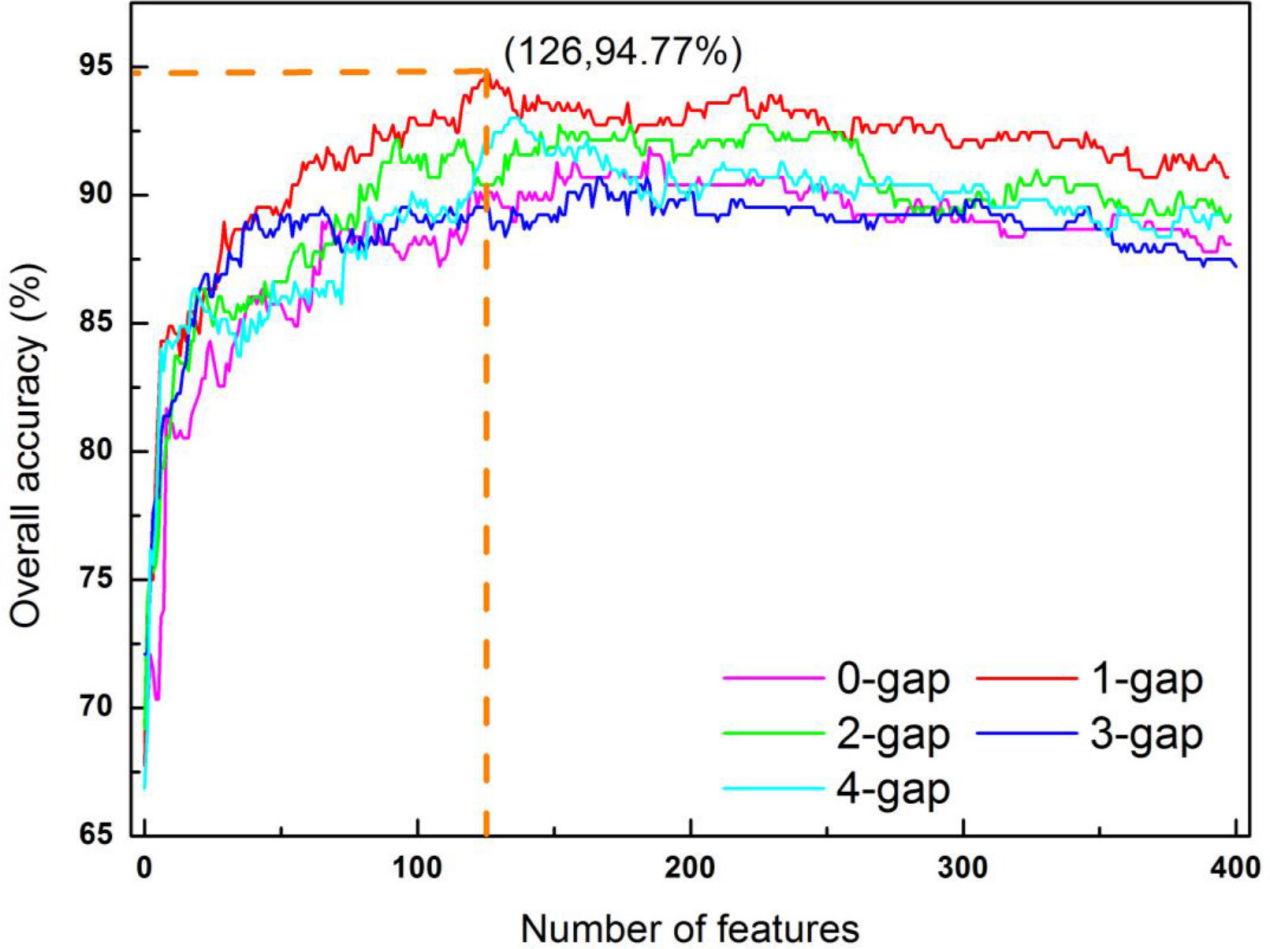
A plot to show the IFS procedure When the top 126 1-gap dipeptides were used to perform prediction, the overall accuracy reached its peak of 94.77%. See the text for more explanation.

As we can see from the Eq.6, the current approach involves five different kinds of dipeptide composition, each containing 400 dipeptide components or corresponding to a 400-D (dimension) vector. Using the feature selection method (see the “Feature Selection” section), the IFS curve was plotted for each of the five different types of dipeptide composition (Figure 4). It can be seen from the figure that when *g* = 1 and Θ = 126, the IFS reaches the peak Acc = 94.77%. Accordingly, the optimal *g*-gap dipeptide set in this study should be *S*_126_ (see Eq.14) derived from one-gap dipeptide composition (see Eq.6). The 126 optimal one-gap dipeptides and their *F*-values (see Eq.10) are listed in Supporting Information S4.

For verifying the advantage of the optimized one-gap dipeptide composition, it is also necessary to investigate the performance of other parameters. Therefore, the amino acid composition and dipeptide composition-based SVM models were developed, respectively. Their jackknife test results in identifying anticancer peptides by using the same benchmark dataset (see Supporting Information S1) are given in Table 3, from which we can see that the performance of the optimized one-gap dipeptide composition is superior to its counterparts.

**Table 3:** A comparison of the current model (iACP) with the other two models via the jackknife tests on the same benchmark dataset (Supporting Information S1)

Parameters	Sn^a^ (%)	Sp^a^ (%)	Acc^a^ (%)	MCC^a^
One-gap dipeptide composition	89.86	98.54	95.06	0.897
Amino acid composition	85.51	94.66	90.99	0.812
Dipeptide composition	72.46	93.69	85.14	0.669

a See the footnote c of Table 1.

The predictor established by going through the above procedures is called iACP, where “i” stands for “identify”, and “ACP” for “anticancer peptide”.

## CONCLUSIONS

The iACP web-server presented here is for identifying whether a peptide belongs to anticancer or non-anticancer purely based on its sequence information alone. The predicted results obtained by iACP via the jackknife test, 5-fold cross-validation test, and independent dataset test have indicated that the new predictor is indeed quite promising, or at the very least, able to play a complimentary role to the existing state-of-the art methods in this area [15, 16]. Owing to its high success rates and user-friendliness, it is anticipated that iACP will become a very useful high throughput tool, being widely used in drug development as well as biomedicine research.

## SUPPLEMENTARY MATERIALS INFORMATION



## References

[1] Ferlay J, Shin HR, Bray F, Forman D, Mathers C, Parkin DM (2010). Estimates of worldwide burden of cancer in 2008. International journal.

[2] Al-Benna S, Shai Y, Jacobsen F, Steinstraesser L (2011). Oncolytic activities of host defense peptides. International journal of molecular sciences.

[3] Kalyanaraman B, Joseph J, Kalivendi S, Wang S, Konorev E, Kotamraju S (2002). Doxorubicin-induced apoptosis: implications in cardiotoxicity. Molecular and cellular biochemistry.

[4] Harris F, Dennison SR, Singh J, Phoenix DA (2013). On the selectivity and efficacy of defense peptides with respect to cancer cells. Medicinal research reviews.

[5] Gaspar D, Veiga AS, Castanho MA (2013). From antimicrobial to anticancer peptides. Frontiers in microbiology.

[6] Huang Y, Feng Q, Yan Q, Hao X, Chen Y (2015). Alpha-helical cationic anticancer peptides: a promising candidate for novel anticancer drugs. Mini reviews in medicinal chemistry.

[7] Chou KC (2004). Review: Structural bioinformatics and its impact to biomedical science. Current Medicinal Chemistry.

[8] Zhang R, Wei DQ, Du QS (2006). Molecular modeling studies of peptide drug candidates against SARS. Medicinal Chemistry.

[9] Wei DQ, Du QS, Sirois S, Zhong WZ (2006). Review: Progress in computational approach to drug development against SARS. Current Medicinal Chemistry.

[10] Mader J, Hoskin D (2006). Cationic antimicrobial peptides as novel cytotoxic agents for cancer treatment. Expert Opin Investig Drugs.

[11] Hoskin DW, Ramamoorthy A (2008). Studies on anticancer activities of antimicrobial peptides. Biochimica et biophysica acta.

[12] Hariharan S, Gustafson D, Holden S, McConkey D, Davis D, Morrow M, Basche M, Gore L, Zang C, O'Bryant CL, Baron A, Gallemann D, Colevas D, Eckhardt SG Assessment of the biological and pharmacological effects of the alpha nu beta3 and alpha nu beta5 integrin receptor antagonist. Annals of oncology.

[13] Gregorc V, De Braud FG, De Pas TM, Scalamogna R, Citterio G, Milani A, Boselli S, Catania C, Donadoni G, Rossoni G, Ghio D, Spitaleri G, Ammannati C (2011). A selective vascular targeting agent in combination with cisplatin in refractory solid tumors. Clinical cancer research.

[14] Thundimadathil J (2012). Cancer treatment using peptides: current therapies and future prospects. Journal of Amino Acids.

[15] Tyagi A, Kapoor P, Kumar R, Chaudhary K, Gautam A, Raghava GP (2013). In silico models for designing and discovering novel anticancer peptides. Scientific Reports.

[16] Hajisharifi Z, Piryaiee M, Mohammad Beigi M, Behbahani M, Mohabatkar H (2014). Predicting anticancer peptides with Chou's pseudo amino acid composition and investigating their mutagenicity via Ames test. Journal of Theoretical Biology.

[17] Chen W, Feng PM, Lin H, Chou KC (2013). iRSpot-PseDNC: identify recombination spots with pseudo dinucleotide composition. Nucleic Acids Res.

[18] Qiu WR, Xiao X, Lin WZ (2014). iMethyl-PseAAC: Identification of Protein Methylation Sites via a Pseudo Amino Acid Composition Approach. Biomed Res Int.

[19] Xu Y, Wen X, Wen LS, Wu LY (2014). iNitro-Tyr: Prediction of nitrotyrosine sites in proteins with general pseudo amino acid composition. PLoS ONE.

[20] Chen W, Feng PM, Deng EZ, Lin H (2014). iTIS-PseTNC: a sequence-based predictor for identifying translation initiation site in human genes using pseudo trinucleotide composition. Anal Biochem.

[21] Guo SH, Deng EZ, Xu LQ, Ding H, Lin H, Chen W (2014). iNuc-PseKNC: a sequence-based predictor for predicting nucleosome positioning in genomes with pseudo k-tuple nucleotide composition. Bioinformatics.

[22] Qiu WR, Xiao X (2014). iRSpot-TNCPseAAC: Identify recombination spots with trinucleotide composition and pseudo amino acid components. Int J Mol Sci.

[23] Liu Z, Xiao X, Qiu WR (2015). iDNA-Methyl: Identifying DNA methylation sites via pseudo trinucleotide composition. Analytical Biochemistry.

[24] Qiu WR, Xiao X, Lin WZ (2015). iUbiq-Lys: Prediction of lysine ubiquitination sites in proteins by extracting sequence evolution information via a grey system model. Journal of Biomolecular Structure and Dynamics.

[25] Jia J, Liu Z, Xiao X (2015). iPPI-Esml: an ensemble classifier for identifying the interactions of proteins by incorporating their physicochemical properties and wavelet transforms into PseAAC. J Theor Biol.

[26] Liu B, Fang L, Liu F, Wang X (2015). Identification of real microRNA precursors with a pseudo structure status composition approach. PLoS ONE.

[27] Chen W, Feng P, Ding H (2015). iRNA-Methyl: Identifying N6-methyladenosine sites using pseudo nucleotide composition. Analytical Biochemistry.

[28] Liu B, Fang L, Wang S, Wang X (2015). Identification of microRNA precursor with the degenerate K-tuple or Kmer strategy. Journal of Theoretical Biology.

[29] Liu B, Fang L, Long R, Lan X (2016). iEnhancer-2L: a two-layer predictor for identifying enhancers and their strength by pseudo k-tuple nucleotide composition. Bioinformatics.

[30] Chou KC (2011). Some remarks on protein attribute prediction and pseudo amino acid composition (50th Anniversary Year Review). J Theor Biol.

[31] Chou KC, Shen HB (2007). Review: Recent progresses in protein subcellular location prediction. Anal Biochem.

[32] Jiang SP, Liu WM, Fee CH (1979). Graph theory of enzyme kinetics: 1. Steady-state reaction system. Scientia Sinica.

[33] Cornish-Bowden A (1979). Fundamentals of Enzyme Kinetics, Chapter 4.

[34] Forsen S (1980). Graphical rules for enzyme-catalyzed rate laws. Biochem J.

[35] Chou KC (1980). A new schematic method in enzyme kinetics. Eur J Biochem.

[36] Liu WM (1981). Graphical rules for non-steady state enzyme kinetics. J Theor Biol.

[37] Zhou GP, Deng MH (1984). An extension of Chou's graphic rules for deriving enzyme kinetic equations to systems involving parallel reaction pathways. Biochem J.

[38] Chou KC (1989). Graphic rules in steady and non-steady enzyme kinetics. J Biol Chem.

[39] Chou KC (1990). Review: Applications of graph theory to enzyme kinetics and protein folding kinetics. Steady and non-steady state systems. Biophysical Chemistry.

[40] Shen HB (2009). FoldRate: A web-server for predicting protein folding rates from primary sequence. The Open Bioinformatics Journal.

[41] Shen HB, Song JN (2009). Prediction of protein folding rates from primary sequence by fusing multiple sequential features. Journal of Biomedical Science and Engineering (JBiSE).

[42] Chou KC, Shen HB (2009). Review: recent advances in developing web-servers for predicting protein attributes. Natural Science.

[43] Althaus IW, Gonzales AJ, Romero DL, Aristoff PA, Tarpley WG, Reusser F (1993). The quinoline U-78036 is a potent inhibitor of HIV-1 reverse transcriptase. J Biol Chem.

[44] Althaus IW, Chou JJ, Gonzales AJ, Diebel MR, Romero DL, Aristoff PA, Tarpley WG, Reusser F (1993). Kinetic studies with the nonnucleoside HIV-1 reverse transcriptase inhibitor U-88204E. Biochemistry.

[45] Althaus IW, Chou JJ, Gonzales AJ, Kezdy FJ, Romero DL, Thomas RC, Aristoff PA, Tarpley WG, Reusser F (1994). Kinetic studies with the non-nucleoside HIV-1 reverse transcriptase inhibitor U-90152E. Biochem Pharmacol.

[46] Diebel MR, Kezdy FJ, Romero DL, Thomas RC, Aristoff PA, Tarpley WG, Reusser F (1996). The benzylthio-pyrididine U-31,355 is a potent inhibitor of HIV-1 reverse transcriptase. Biochem Pharmacol.

[47] Kezdy FJ, Reusser F (1994). Review: Steady-state inhibition kinetics of processive nucleic acid polymerases and nucleases. Anal Biochem.

[48] Forsen S (1981). Graphical rules of steady-state reaction systems. Can J Chem.

[49] Chou KC (2010). Graphic rule for drug metabolism systems. Current Drug Metabolism.

[50] Chou KC, Zhang CT (1992). Diagrammatization of codon usage in 339 HIV proteins and its biological implication. AIDS Research and Human Retroviruses.

[51] Zhang CT (1993). Graphic analysis of codon usage strategy in 1490 human proteins. J Protein Chem.

[52] Zhang CT, Chou KC (1994). Analysis of codon usage in 1562 E. Coli protein coding sequences. J Mol Biol.

[53] Chou KC, Zhang CT, Elrod DW (1996). Do antisense proteins exist?. J Protein Chem.

[54] Wu ZC, Xiao X (2010). 2D-MH: A web-server for generating graphic representation of protein sequences based on the physicochemical properties of their constituent amino acids. J Theor Biol.

[55] Chou KC, Lin WZ, Xiao X (2011). Wenxiang: a web-server for drawing wenxiang diagrams. Natural Science.

[56] Zhou GP (2011). The disposition of the LZCC protein residues in wenxiang diagram provides new insights into the protein-protein interaction mechanism. J Theor Biol.

[57] Zhou GP, Huang RB (2013). The pH-Triggered Conversion of the PrP(c) to PrP(sc.). Curr Top Med Chem.

[58] Wilkinson L, Friendly M (2009). The history of the cluster heat map. The American Statistician.

[59] Dobrzynska I, Szachowicz-Petelska B, Sulkowski S, Figaszewski Z (2005). Changes in electric charge and phospholipids composition in human colorectal cancer cells. Molecular and cellular biochemistry.

[60] Utsugi T, Schroit AJ, Connor J, Bucana CD, Fidler IJ (1991). Elevated expression of phosphatidylserine in the outer membrane leaflet of human tumor cells and recognition by activated human blood monocytes. Cancer research.

[61] Huang YB, Wang XF, Wang HY, Liu Y, Chen Y (2011). Studies on mechanism of action of anticancer peptides by modulation of hydrophobicity within a defined structural framework. Molecular cancer therapeutics.

[62] Wang G, Li X, Wang Z (2009). APD2: the updated antimicrobial peptide database and its application in peptide design. Nucleic acids research.

[63] Bals R (2000). Epithelial antimicrobial peptides in host defense against infection. Respiratory research.

[64] Fu L, Niu B, Zhu Z, Wu S, Li W (2012). CD-HIT: accelerated for clustering the next-generation sequencing data. Bioinformatics.

[65] Chou KC, Wu ZC, Xiao X (2012). iLoc-Hum: Using accumulation-label scale to predict subcellular locations of human proteins with both single and multiple sites. Molecular Biosystems.

[66] Tyagi A, Tuknait A, Anand P, Gupta S, Sharma M, Mathur D, Joshi A, Singh S, Gautam A, Raghava GP (2015). CancerPPD: a database of anticancer peptides and proteins. Nucleic Acids Res.

[67] Wootton JC, Federhen S (1993). Statistics of local complexity in amino acid sequences and sequence databases. Comput Chem.

[68] Altschul SF, Madden TL, Schaffer AA, Zhang J, Zhang Z, Miller W, Lipman DJ (1997). Gapped BLAST and PSI-BLAST: a new generation of protein database search programs. Nucleic Acids Res.

[69] Zhang CT (1992). An optimization approach to predicting protein structural class from amino acid composition. Protein Science.

[70] Chou KC, Zhang CT (1992). A correlation coefficient method to predicting protein structural classes from amino acid compositions. Eur J Biochem.

[71] Chou JJ (1993). A formulation for correlating properties of peptides and its application to predicting human immunodeficiency virus protease-cleavable sites in proteins. Biopolymers.

[72] Thompson TB, Zheng C (1995). Neural network prediction of the HIV-1 protease cleavage sites. Journal of Theoretical Biology 177.

[73] Zhou GP, Doctor K (2003). Subcellular location prediction of apoptosis proteins. Proteins: Struct, Funct, Genet.

[74] Chou KC (2005). Prediction of G-protein-coupled receptor classes. Journal of Proteome Research.

[75] Wang M, Yang J, Xu ZJ (2005). SLLE for predicting membrane protein types. J Theor Biol.

[76] Shen HB (2005). Using optimized evidence-theoretic K-nearest neighbor classifier and pseudo amino acid composition to predict membrane protein types. Biochemical & Biophysical Research Communications.

[77] Chou KC, Shen HB (2007). Euk-mPLoc: a fusion classifier for large-scale eukaryotic protein subcellular location prediction by incorporating multiple sites. Journal of Proteome Research.

[78] Xiao X, Wang P (2011). GPCR-2L: Predicting G protein-coupled receptors and their types by hybridizing two different modes of pseudo amino acid compositions. Molecular Biosystems.

[79] Wang P, Xiao X (2011). NR-2L: A Two-Level Predictor for Identifying Nuclear Receptor Subfamilies Based on Sequence-Derived Features. PLoS ONE.

[80] Kandaswamy KK, Moller S, Suganthan PN, Sridharan S, Pugalenthi G (2011). AFP-Pred: A random forest approach for predicting antifreeze proteins from sequence-derived properties. J Theor Biol.

[81] Xu Y, Ding J, Wu LY (2013). iSNO-PseAAC: Predict cysteine S-nitrosylation sites in proteins by incorporating position specific amino acid propensity into pseudo amino acid composition. PLoS ONE.

[82] Chou KC (2013). Some Remarks on Predicting Multi-Label Attributes in Molecular Biosystems. Molecular Biosystems.

[83] Liu B, Zhang D, Xu R, Xu J, Wang X (2014). Combining evolutionary information extracted from frequency profiles with sequence-based kernels for protein remote homology detection. Bioinformatics.

[84] Nakashima H, Nishikawa K, Ooi T (1986). The folding type of a protein is relevant to the amino acid composition. J Biochem.

[85] Klein P, Delisi C (1986). Prediction of protein structural class from amino acid sequence. Biopolymers.

[86] Cedano J, Aloy P, Perez-Pons JA, Querol E (1997). Relation between amino acid composition and cellular location of proteins. J Mol Biol.

[87] Zhou GP (1998). An intriguing controversy over protein structural class prediction. J Protein Chem.

[88] Chou KC (2001). Prediction of protein cellular attributes using pseudo amino acid composition. PROTEINS.

[89] Chou KC (2005). Using amphiphilic pseudo amino acid composition to predict enzyme subfamily classes. Bioinformatics.

[90] Lin SX, Lapointe J (2013). Theoretical and experimental biology in one —A symposium in honour of Professor Kuo-Chen Chou's 50th anniversary and Professor Richard Giegé's 40th anniversary of their scientific careers. J Biomedical Science and Engineering.

[91] Cao DS, Xu QS, Liang YZ (2013). propy: a tool to generate various modes of Chou's PseAAC. Bioinformatics.

[92] Du P, Gu S, Jiao Y (2014). PseAAC-General: Fast building various modes of general form of Chou's pseudo-amino acid composition for large-scale protein datasets. International Journal of Molecular Sciences.

[93] Zhou XB, Chen C, Li ZC, Zou XY (2007). Using Chou's amphiphilic pseudo-amino acid composition and support vector machine for prediction of enzyme subfamily classes. J Theor Biol.

[94] Esmaeili M, Mohabatkar H, Mohsenzadeh S (2010). Using the concept of Chou's pseudo amino acid composition for risk type prediction of human papillomaviruses. J Theor Biol.

[95] Sahu SS, Panda G (2010). A novel feature representation method based on Chou's pseudo amino acid composition for protein structural class prediction. Computational Biology and Chemistry.

[96] Mohabatkar H, Mohammad Beigi M, Esmaeili A (2011). Prediction of GABA(A) receptor proteins using the concept of Chou's pseudo-amino acid composition and support vector machine. J Theor Biol.

[97] Mohammad Beigi M, Behjati M, Mohabatkar H (2011). Prediction of metalloproteinase family based on the concept of Chou's pseudo amino acid composition using a machine learning approach. Journal of Structural and Functional Genomics.

[98] Nanni L, Lumini A, Gupta D, Garg A (2012). Identifying bacterial virulent proteins by fusing a set of classifiers based on variants of Chou's pseudo amino acid composition and on evolutionary information. IEEE-ACM Transaction on Computational Biolology and Bioinformatics.

[99] Gupta MK, Niyogi R, Misra M (2013). An alignment-free method to find similarity among protein sequences via the general form of Chou's pseudo amino acid composition. SAR QSAR Environ Res.

[100] Hajisharifi Z, Piryaiee M, Mohammad Beigi M, Behbahani M, Mohabatkar H (2014). Predicting anticancer peptides with Chou's pseudo amino acid composition and investigating their mutagenicity via Ames test. J Theor Biol.

[101] Huang C, Yuan JQ (2013). Predicting protein subchloroplast locations with both single and multiple sites via three different modes of Chou's pseudo amino acid compositions. J Theor Biol.

[102] Mohabatkar H, Beigi MM, Abdolahi K, Mohsenzadeh S (2013). Prediction of Allergenic Proteins by Means of the Concept of Chou's Pseudo Amino Acid Composition and a Machine Learning Approach. Medicinal Chemistry.

[103] Khan ZU, Hayat M, Khan MA (2015). Discrimination of acidic and alkaline enzyme using Chou's pseudo amino acid composition in conjunction with probabilistic neural network model. J Theor Biol.

[104] Dehzangi A, Heffernan R, Sharma A, Lyons J, Paliwal K, Sattar A (2015). Gram-positive and Gram-negative protein subcellular localization by incorporating evolutionary-based descriptors into Chou's general PseAAC. J Theor Biol.

[105] Kumar R, Srivastava A, Kumari B, Kumar M (2015). Prediction of beta-lactamase and its class by Chou's pseudo-amino acid composition and support vector machine. J Theor Biol.

[106] Wang X, Zhang W, Zhang Q, Li GZ (2015). MultiP-SChlo: multi-label protein subchloroplast localization prediction with Chou's pseudo amino acid composition and a novel multi-label classifier. Bioinformatics.

[107] Mandal M, Mukhopadhyay A, Maulik U (2015). Prediction of protein subcellular localization by incorporating multiobjective PSO-based feature subset selection into the general form of Chou's PseAAC. Medical & biological engineering & computing.

[108] Liu B, Liu F, Wang X, Chen J (2015). Chou KC. Pse-in-One: a web server for generating various modes of pseudo components of DNA, RNA, and protein sequences Nucleic Acids Res.

[109] Chen W, Lin H, Chou KC (2015). Pseudo nucleotide composition or PseKNC: an effective formulation for analyzing genomic sequences. Mol BioSyst.

[110] Zhong WZ, Zhou SF (2014). Molecular science for drug development and biomedicine. Intenational Journal of Molecular Sciences.

[111] Chou KC (2015). Impacts of bioinformatics to medicinal chemistry. Medicinal Chemistry.

[112] Xu Y (2016). Recent progress in predicting posttranslational modification sites in proteins. Curr Top Med Chem.

[113] Chen W, Lin H (2012). Identification of voltage-gated potassium channel subfamilies from sequence information using support vector machine. Computers in biology and medicine.

[114] Lin H, Chen W (2011). Prediction of thermophilic proteins using feature selection technique. Journal of microbiological methods.

[115] Ding H, Deng EZ, Yuan LF, Liu L (2014). iCTX-Type: A Sequence-Based Predictor for Identifying the Types of Conotoxins in Targeting Ion Channels. BioMed research international.

[116] Ding H, Feng PM, Chen W, Lin H (2014). Identification of bacteriophage virion proteins by the ANOVA feature selection and analysis. Molecular bioSystems.

[117] Lin H, Chen W, Ding H (2013). AcalPred: a sequence-based tool for discriminating between acidic and alkaline enzymes. PloS one.

[118] Liu W (1999). Protein secondary structural content prediction. Protein Eng.

[119] Xu Y, Wen X, Shao XJ (2014). iHyd-PseAAC: Predicting hydroxyproline and hydroxylysine in proteins by incorporating dipeptide position-specific propensity into pseudo amino acid composition. Int J Mol Sci.

[120] Xu Y, Shao XJ, Wu LY, Deng NY (2013). iSNO-AAPair: incorporating amino acid pairwise coupling into PseAAC for predicting cysteine S-nitrosylation sites in proteins. PeerJ.

[121] Han GS, Yu ZG, Anh V (2014). A two-stage SVM method to predict membrane protein types by incorporating amino acid classifications and physicochemical properties into a general form of Chou's PseAAC. J Theor Biol.

[122] Lin H, Deng EZ, Ding H, Chen W, Chou KC (2014). iPro54-PseKNC: a sequence-based predictor for identifying sigma-54 promoters in prokaryote with pseudo k-tuple nucleotide composition. Nucleic Acids Res.

[123] Xiao X, Min JL, Lin WZ, Liu Z (2015). iDrug-Target: predicting the interactions between drug compounds and target proteins in cellular networking via the benchmark dataset optimization approach. Journal of Biomolecular Structure & Dynamics.

[124] Liu B, Fang L, Liu F, Wang X (2016). iMiRNA-PseDPC: microRNA precursor identification with a pseudo distance-pair composition approach. Journal of Biomolecular Structure & Dynamics.

[125] Chou KC, Cai YD (2002). Using functional domain composition and support vector machines for prediction of protein subcellular location. J Biol Chem.

[126] Cai YD, Zhou GP (2003). Support vector machines for predicting membrane protein types by using functional domain composition. Biophys J.

[127] Cristianini N, Shawe-Taylor J (2000). An introduction of Support Vector Machines and other kernel-based learning methodds.

[128] Chang C, Lin CJ (2001). LIBSVM: A Library for Support Vector Machines. ACM Transactions on Intelligent Systems and Technology.

[129] Chou KC, Zhang CT (1995). Review: Prediction of protein structural classes. Crit Rev Biochem Mol Biol.

[130] Chou KC, Cai YD (2005). Prediction of membrane protein types by incorporating amphipathic effects. Journal of Chemical Information and Modeling.

[131] Shen HB (2007). Virus-PLoc: A fusion classifier for predicting the subcellular localization of viral proteins within host and virus-infected cells. Biopolymers.

[132] Chen W, Lin H, Feng PM, Ding C (2012). iNuc-PhysChem: A Sequence-Based Predictor for Identifying Nucleosomes via Physicochemical Properties. PLoS ONE.

[133] Sun XY, Shi SP, Qiu JD, Suo SB, Huang SY, Liang RP (2012). Identifying protein quaternary structural attributes by incorporating physicochemical properties into the general form of Chou's PseAAC via discrete wavelet transform. Molecular BioSystems.

[134] Kabir M, Hayat M (2016). iRSpot-GAEnsC: identifing recombination spots via ensemble classifier and extending the concept of Chou's PseAAC to formulate DNA samples. Molecular genetics and genomics.

[135] Chou KC (2001). Using subsite coupling to predict signal peptides. Protein Eng.

[136] Jia J, Liu Z, Xiao X, Liu B (2016). iPPBS-Opt: A Sequence-Based Ensemble Classifier for Identifying Protein-Protein Binding Sites by Optimizing Imbalanced Training Datasets. Molecules.

[137] Jia J, Liu Z, Xiao X, Liu B (2016). iSuc-PseOpt: Identifying lysine succinylation sites in proteins by incorporating sequence-coupling effects into pseudo components and optimizing imbalanced training dataset. Anal Biochem.

[138] Liu Z, Xiao X, Yu DJ, Jia J, Qiu WR (2016). pRNAm-PC: Predicting N-methyladenosine sites in RNA sequences via physical-chemical properties. Anal Biochem.

[139] Chen W, Feng P, Ding H, Lin H (2016). Using deformation energy to analyze nucleosome positioning in genomes. Genomics.

[140] Chou KC (1993). A vectorized sequence-coupling model for predicting HIV protease cleavage sites in proteins. J Biol Chem.

[141] Lin WZ, Fang JA, Xiao X (2013). iLoc-Animal: A multi-label learning classifier for predicting subcellular localization of animal proteins. Molecular BioSystems.

[142] Xiao X, Wu ZC (2011). iLoc-Virus: A multi-label learning classifier for identifying the subcellular localization of virus proteins with both single and multiple sites. J Theor Biol.

[143] Xiao X, Wang P, Lin WZ (2013). iAMP-2L: A two-level multi-label classifier for identifying antimicrobial peptides and their functional types. Anal Biochem.

[144] Lin H, Ding H (2011). Predicting ion channels and their types by the dipeptide mode of pseudo amino acid composition. J Theor Biol.

[145] Li BQ, Hu LL, Chen L, Feng KY (2012). Prediction of Protein Domain with mRMR Feature Selection and Analysis. PLoS One.

[146] Wang P, Hu L, Liu G, Jiang N, Chen X, Xu J, Zheng W, Li L, Tan M, Chen Z (2011). Prediction of antimicrobial peptides based on sequence alignment and feature selection methods. PLoS ONE.

[147] Li BQ, Huang T, Liu L (2012). Identification of colorectal cancer related genes with mRMR and shortest path in protein-protein interaction network. PLoS ONE.

[148] Huang T, Wang J, Cai YD (2012). Hepatitis C virus network based classification of hepatocellular cirrhosis and carcinoma. PLoS ONE.

