# Supplementary material for: iACP: a sequence-based tool for identifying anticancer peptides
Source: Oncotarget. 2016 Mar 1;7(13):16895–909. doi: 10.18632/oncotarget.7815 (PMC4941358; doi:10.18632/oncotarget.7815)
Supplement: Supplementary file 1 [file oncotarget-07-16895-s001.pdf]

# iACP: a sequence-based tool for identifying anticancer peptides

## Supplementary Materials

### Supporting Information S1: The benchmark dataset contains 138 anticancer peptides and 206 non-anticancer peptides (see Eq.1)

**I. 138 anticancer peptides**

| |
|---|
| > ACP_1 |
| GLWSKIKEVGKEAAKAAAKAAGKAALGAVSEAV |
| > ACP_2 |
| GLFDIIKKIAESI |
| > ACP_3 |
| GLLDIVKKVVGAFGSL |
| > ACP_4 |
| GLFDIVKKVVGALGSL |
| > ACP_5 |
| GLFDIVKKVVGTLAGL |
| > ACP_6 |
| GLFDIAKKVIGVIGSL |
| > ACP_7 |
| GLFDIVKKIAGHIAGSI |
| > ACP_8 |
| GLFDIVKKIAGHIVSSI |
| > ACP_9 |
| AACARFIDDFCDTLTPNIYRPRDNGQRCYAVNGHRCDFTVFNTNNGGNPIRASTPN CKTVLRTAANRCPTGGRGKINPNAPFLFAIDPNDGDCSTNF |
| > ACP_10 |
| HGVSGHGQHGVHG |
| > ACP_11 |
| FKCRRWQWRMKKLGAPSITCVRRAF |
| > ACP_12 |
| KWKLFKKIKFLHSAKKF |
| > ACP_13 |
| KSSAYSLQMGATAIKQVKKLFKKWGW |
| > ACP_14 |
| GIGTKILGGVKTALKGALKELASTYAN |
| > ACP_15 |
| GIGGKILSGLKTALKGAAKELASTYLH |
| > ACP_16 |
| GIGGVLLSAGKAALKGLAKVLAEKYAN |
| > ACP_17 |
| SIGAKILGGVKTFFKGALKELASTYLQ |
| > ACP_18 |
| FLPLLAGLAANFLPTIICKISYKC |

| |
|---|
| > ACP_19 |
| FVQWFSKFLGRIL |
| > ACP_20 |
| KWKIFKKIEKVGRNIRNGIIKAGPAVAVLGEAKAL |
| > ACP_21 |
| GWLKKIGKKIERVGQHTRDATIQTIGVAQQAANVAATLK |
| > ACP_22 |
| KWKLFKKIEKVGQNIRDGIIKAGPAVAVVGQATQIAK |
| > ACP_23 |
| SQLGDLGSGAGQGGGGGGSIRAAGGAFGKLEAAREEEFFYKKQKEQLERLKND<br>QIHQAEFHHQQIKEHEEAIQRHKDFLNNLHK |
| > ACP_24 |
| GIGKFLHSAKKFGKAFVGEIMNS |
| > ACP_25 |
| GIGAVLKVLTTGLPALISWIKRKRQQ |
| > ACP_26 |
| ALWKNMLKGIGKLAGQAALGAVKTLVGAE |
| > ACP_27 |
| ACYCRIPACIAGERRYGTCIYQGRLWAFCC |
| > ACP_28 |
| ECRRLCYKQRCVTYCRGR |
| > ACP_29 |
| LKLKSIVSWAKKVL |
| > ACP_30 |
| KWCFRVCYRGICYRRCR |
| > ACP_31 |
| KSCCRNTWARNCYNVCRLPGTISREICAKKCDCKIISGTTCPSDYPK |
| > ACP_32 |
| GLLSVLGSVAKHVLPHVVPVIAEHL |
| > ACP_33 |
| GLLSVLGSVVKHVIPHVVPVIAEHL |
| > ACP_34 |
| GLFKVLGSVAKHLLPHVAPVIAEK |
| > ACP_35 |
| GLFGVLGSIAKHVLPHVVPVIAEK |
| > ACP_36 |
| GLFVGVLAKVAAHVVPAIAEHF |
| > ACP_37 |
| GLFVGLAKVAAHNNPAIAEHFQA |
| > ACP_38 |
| GFVDFLKKVAGTIANVVT |
| > ACP_39 |
| GLLQTIKEKLESLESLAKGIVSGIQA |
| > ACP_40 |
| TRSSRAGLQFPVGRVHRLLRK |
| > ACP_41 |
| FFGWLIKGAIHAGKAIHGLIHRRRH |
| > ACP_42 |

| |
|---|
| GLFDVIKKVASVIGGL |
| > ACP_43 |
| GLFDIIKKVASVVGGL |
| > ACP_44 |
| GRFKRFRKKFKKLFKKLSPVIPLLHLG |
| > ACP_45 |
| GGLRSLGRKILRAWKKYGPIIVPIIRIG |
| > ACP_46 |
| RRRPRPPYLPRPRPPPFFPPRLPPRIPPGFPPRFPPRFP |
| > ACP_47 |
| GLLGPLLKIAAKVGSNLL |
| > ACP_48 |
| GLICESCRKIIQKLEDMVGPQPNEDTVTQAASQVCDKLKILRGLCKKIMRSFLRRISWDILTG<br>KKPQAICVDIKICKEKTGLI |
| > ACP_49 |
| DHYNCVSSGGQCLYSACPIFTKIQGTCYRGKAKCCK |
| > ACP_50 |
| FFHHIFRGIVHVGKTIHRLVTG |
| > ACP_51 |
| KLAKLAKKLAKLAK |
| > ACP_52 |
| KTCENLADTFRGPCFATSNC |
| > ACP_53 |
| IDWKKLLDAAKQIL |
| > ACP_54 |
| FLIGMTQGLICLITRKC |
| > ACP_55 |
| ILPILSLIGGLLGK |
| > ACP_56 |
| GLLGLLGSVVSHVVPAIVGHF |
| > ACP_57 |
| GLLGLLGSVVSHVLPAITQHL |
| > ACP_58 |
| GIKCRFCCGCCTPGICGVCCRF |
| > ACP_59 |
| QSHLSLCRWCCNCCRSNKGC |
| > ACP_60 |
| ILGPVISTIGGVLGGLLKNL |
| > ACP_61 |
| FLPILASLAAKFGPKLFCLVTKKC |
| > ACP_62 |
| GLWSKIKEAAKAAGKAALNAVTGLVNQGDQPS |
| > ACP_63 |
| LLGMIPLAISAISALSKL |
| > ACP_64 |
| GLPVCGETCVGGTCNTPGCSCSWPVCTRN |
| > ACP_65 |
| GVPICGETCTLGTCYTAGCSCSWPVCTRN |

| |
|---|
| > ACP_66 |
| GIPCGESCVWIPCISSAIGCSCKSKVCYRN |
| > ACP_67 |
| GIPCAESCVWIPCTVTALIGCGCSNKVCYN |
| > ACP_68 |
| GTFPCGESCVFIPCLTSAIGCSCKSKVCYKN |
| > ACP_69 |
| GLLPCAESCVYIPCLTTVIGCSCKSKVCYKN |
| > ACP_70 |
| GRDYRTCLTIVQKLKKMVDKPTQRSVSNAATRVCRTGRSRWRDVCRNFMRRYQSRVTQGLVAGE<br>TAQQICEDLRLCIPSTGPL |
| > ACP_71 |
| GETDPNTQLLNDLGNNMAWGAALGAPGGLGSAALGAAGGALQTVGQGLIDHGPVNVFIPVLIGPSW<br>NGSGSGYNSATSSSGSGS |
| > ACP_72 |
| GFKDLLKGAAKALVKTVLF |
| > ACP_73 |
| KSCCPNTTGRNIYNACRLTGAPRPTCAKLSGCKIISGSTCPSDYPK |
| > ACP_74 |
| KSCCPNTTGRNIYNTCRFGGGSREVCARISGCKIISASTCPSDYPK |
| > ACP_75 |
| KSCCPNTTGRNIYNTCRLTGSSRETCAKLSGCKIISASTCPSNYPK |
| > ACP_76 |
| MRKEFHNVLSSGQLLADKRPARDYNRK |
| > ACP_77 |
| KSCCKNTTGRNIYNTCRFAGGSRERCAKLSGCKIISASTCPSDYPK |
| > ACP_78 |
| FIFHIIKGLFHAGKMIHGLVTRRRH |
| > ACP_79 |
| FLPAIVGAAAKFLPKIFCAISKKC |
| > ACP_80 |
| FLPIIAGVAAKVLPKIFCAISKKC |
| > ACP_81 |
| FLPIIAGIAAKFLPKIFCTISKKC |
| > ACP_82 |
| FLPVIAGVAANFLPKLFCAISKKC |
| > ACP_83 |
| FLPIIAGAAAKVVQKIFCAISKKC |
| > ACP_84 |
| GLMDTIKGVAKTVAASWLDKLKCKITGC |
| > ACP_85 |
| VNWKKVLGKIIKVAK |
| > ACP_86 |
| VNWKKILGKIIKVAK |
| > ACP_87 |
| FFSLLPSLIGGLVSAIK |
| > ACP_88 |

| |
|---|
| RFRLPFRRPPIRIHPPPFYPPFRRFL |
| > ACP_89 |
| KWKLFKKIPKFLHLAKKF |
| > ACP_90 |
| YKQCHKKGGHCFPKEKICLPPSSDFGKMDCRWRWKCCKKGSG |
| > ACP_91 |
| GIPCGESCVFIPCITGAIGCSCKSKVCYRN |
| > ACP_92 |
| GEFLKCGESCVQGECYTPGCSCDWPICKKN |
| > ACP_93 |
| GLPTCGETCTLGTCYVPDCSCSWPICMKN |
| > ACP_94 |
| GLPVCGETCFGGTCNTPGCTCDPWPVCTRN |
| > ACP_95 |
| FVDLKKIANIINSIFGK |
| > ACP_96 |
| GSIPCGESCVFIPCISSVIGCACKSKVCYKN |
| > ACP_97 |
| GIPCGESCVFIPCISSVIGCSCSSKVCYRN |
| > ACP_98 |
| GSIPCGESCVFIPCISAVIGCSCSNKVCYKN |
| > ACP_99 |
| GSIPCEGSCVFIPCISAIIGCSCSNKVCYKN |
| > ACP_100 |
| GIPCGESCVFIPCLTSAIDCSCKSKVCYRN |
| > ACP_101 |
| GMWSKILGHLIR |
| > ACP_102 |
| GKWMSLLKHILK |
| > ACP_103 |
| GFGMALKLLKKVL |
| > ACP_104 |
| GTGLPMSERRKIMLMMR |
| > ACP_105 |
| GIACGESCVFLGCFIPGCSCKSKVCYFN |
| > ACP_106 |
| GVIPCGESCVFIPCISSVLGCSCKNKVCYRD |
| > ACP_107 |
| KLCGETCFKFKCYTPGCSCSYPFCK |
| > ACP_108 |
| GDACGETCFTGICFTAGCSCNPWPTCTRN |
| > ACP_109 |
| GIPCAESCVWIPPCTITALMGCSCKNNVCYNN |
| > ACP_110 |
| IPCGESCVWIPCITAIAGCSCKNKVCYT |
| > ACP_111 |
| AIPCGESCVWIPCISTVIGCSCSNKVCYR |

| |
|---|
| > ACP_112 |
| GEYCGESCYLIPCFTPGCYCVSRQCVNKN |
| > ACP_113 |
| IPCGESCVWIPCISGMFGCSCKDKVCYS |
| > ACP_114 |
| FLGWLFKWASK |
| > ACP_115 |
| FLKWLFKWAKK |
| > ACP_116 |
| KWKSFLKTFKSAKKTVLHTALKAISS |
| > ACP_117 |
| KWKSFLKTFKSLKKTVLHTLLKAISS |
| > ACP_118 |
| MPFLFCNVNDVCNFASRNDYSCNYYSNSYSFWLASLNPER |
| > ACP_119 |
| KWKLFKKIGAVLKVL |
| > ACP_120 |
| GACFSIAHECGA |
| > ACP_121 |
| TCCATGACGTTCCTGACGTT |
| > ACP_122 |
| KRFKQDGGASHASPASS |
| > ACP_123 |
| KRAKAAGGWSHWSPWSSC |
| > ACP_124 |
| LLGDFFRKSKEKIGKEFKRIVQRIKDFLRNLVPRTES |
| > ACP_125 |
| FLGALFKVASKVLPSVKCAITKKC |
| > ACP_126 |
| GIGKFLKKAKKFGKAFVKILKK |
| > ACP_127 |
| GIGKFLKKAKKGIGAVLKVLTTGL |
| > ACP_128 |
| VECYGPNRPQF |
| > ACP_129 |
| KRFKQDGGWSHWSPWSSC |
| > ACP_130 |
| RQVFQVAYIIIKA |
| > ACP_131 |
| KAFDITYVRLKF |
| > ACP_132 |
| DFKLFAVTIKYR |
| > ACP_133 |
| DFKLFAVYIKYR |
| > ACP_134 |
| WHSDMEWWYLLG |
| > ACP_135 |

| |
|---|
| HTMYYHHYQHHL |
| > ACP_136 |
| RLVSYNGIIFFLK |
| > ACP_137 |
| GRENYHGCTTHWGFTLC |
| > ACP_138 |
| ASSSYPLIHWRPWAR |
| **II. 206 non-anticancer peptides** |
| > non-ACP_1 |
| MTISLIWGIAMVVCCCIWVIFDRRRRKAGEPPL |
| > non-ACP_2 |
| MFATPLRQPTNASGARPAVSMDGQETPFQYEITD |
| > non-ACP_3 |
| LLWRKVAGATVGPGPVPA |
| > non-ACP_4 |
| DSPDPMNGASSNALIAKMNSAKLLYQHY |
| > non-ACP_5 |
| NNQEVIDAISQAISQTPGCVL |
| > non-ACP_6 |
| KKVVEKNADPETTLLVYLRRKLGLCGTKLGCGEG |
| > non-ACP_7 |
| CSRLLPSLAQEEG |
| > non-ACP_8 |
| KNDFAALQAKLDADAAEIEKWWSDSR |
| > non-ACP_9 |
| VDREQLVQKARLAEQAERYDD |
| > non-ACP_10 |
| RPLRRVVLFYQGKLCSMAGNFWQSSHYLQW |
| > non-ACP_11 |
| GAAGERKLCLLSLLLIGA |
| > non-ACP_12 |
| MFRKLLKMWILLRPTHWLILIALCAVTCAGYWLLWSE |
| > non-ACP_13 |
| HLRGPADSGWMPQAAPCLSGAPQAS |
| > non-ACP_14 |
| XSARLTVLLRHLGCRSAGTI |
| > non-ACP_15 |
| NNPNNSNSHLRPHAYNNSRRDDSD |
| > non-ACP_16 |
| VVILASLSVMFLVSLWQQKIRERLPPGPTPLPFIGNY |
| > non-ACP_17 |
| ICLSCLISFFLWNQNRAKGKLPPG |
| > non-ACP_18 |
| VVMNSLRVILQAS |
| > non-ACP_19 |
| ARPRLDLQLVQRFVRIQKVF |
| > non-ACP_20 |

| |
|---|
| MLAKGLSLRSVLAKGCQPFLSPTWQSSVLATGGGANIS |
| > non-ACP_21 |
| AAGLPGAALPLRKRPLRAPSPEPAAPRGAAGLVV |
| > non-ACP_22 |
| PPMPSAPPVHPPP |
| > non-ACP_23 |
| SCPIDKRRPLIAFLRRLRD |
| > non-ACP_24 |
| RLGLWASGLILILGFLKLLRLLLRRQRLARAMD |
| > non-ACP_25 |
| FSPQRDRFQAEGS |
| > non-ACP_26 |
| GTLWALVFLGILVGMVVPSPAGTRANNTLLDSRG |
| > non-ACP_27 |
| MNRLLQKGTSLVPSWRTR |
| > non-ACP_28 |
| MTTSLIWGIAIAACCCLWLILGIRRRQT |
| > non-ACP_29 |
| ATLANGMSLQPPLEEVS |
| > non-ACP_30 |
| PLTATNSGLAVNN |
| > non-ACP_31 |
| VRACHKVCRCLLSGFGGRVDAGQPELLTER |
| > non-ACP_32 |
| TAGILLLLLLGTLEGS |
| > non-ACP_33 |
| MEPSILLLLALLVGFLLLLVRGH |
| > non-ACP_34 |
| MKNCFQLLCNLKVPAAGFKNTVKS |
| > non-ACP_35 |
| SVIHTQAHASGLQQVPQLVPAGPGGGGKAVAPSKQSK |
| > non-ACP_36 |
| KPLGLLKPSSLMKVSGRFKAHQDA |
| > non-ACP_37 |
| ARTLNNKLSLSKPKFSGFT |
| > non-ACP_38 |
| LLLVIIWTLFGPSGLGEELLSLSLASLLPAPASPGPP |
| > non-ACP_39 |
| WPGILVGGARVASCRYPALGPRLA |
| > non-ACP_40 |
| RSVKGLVALITGGASGL |
| > non-ACP_41 |
| AAAALRARILQVSSKVN |
| > non-ACP_42 |
| TGCCIAGRLANLDDQNLTVAL |
| > non-ACP_43 |
| GSILGFLQIATVLTVLLLLLK |

| |
|---|
| > non-ACP_44 |
| AARQIGSCLMRCRTLDTTSP |
| > non-ACP_45 |
| WGCRGRRWAFARVDGGSCHRRGAPTGSTSNQIR |
| > non-ACP_46 |
| YAKPGAVRSPAQILQWQVLPNTVPAKS |
| > non-ACP_47 |
| RMAGPWLSLHEARLLGTRGAAAPKAV |
| > non-ACP_48 |
| SISNRAAVPEHGVAPDAERL |
| > non-ACP_49 |
| PNFSMETWLLLV |
| > non-ACP_50 |
| PRPPSKTYRGAFQN |
| > non-ACP_51 |
| SVLVKGCQPFLSAPRECPGHPRVGT |
| > non-ACP_52 |
| LVTPPKALLKPLSIPNQ |
| > non-ACP_53 |
| KMQGSRMDEQRCS |
| > non-ACP_54 |
| VIADDLPPTCIRP |
| > non-ACP_55 |
| LPGGLRVLVQTGH |
| > non-ACP_56 |
| GWIWRWGWGRRCLGRPGLPGPGPGPATPLFLLLL |
| > non-ACP_57 |
| RGIRGSSAARPSGRRRDPAGRTTETGFNIFTQHD |
| > non-ACP_58 |
| QQEKEFLESYPQNCPPDALPGTPGNLD |
| > non-ACP_59 |
| APARRVLQVKRVMQESSLSPAHL |
| > non-ACP_60 |
| KVAPGGPTGYPGNLTAEQEQKLGELKMILL |
| > non-ACP_61 |
| FLASYPQKCPAGSLPGTPGNTDE |
| > non-ACP_62 |
| MDAKARNCLLQHREALEKDIKTSY |
| > non-ACP_63 |
| ASRQLLVAPPEAL |
| > non-ACP_64 |
| MISNGIGTVTTGKRSMCLFPLLLIGLWGC |
| > non-ACP_65 |
| MTLRNFGMGKRSIEDRVQEEARCLVEELRKTNASPC |
| > non-ACP_66 |
| AVFGLGGVGLSVIMGCKAAGASRIIAVDIN |
| > non-ACP_67 |

| |
|---|
| PNAKQSILQKNPDDVVIVAAYRTA |
| > non-ACP_68 |
| AMELLLTATIFYLVLWVVKAFRLQVPKGLKSPPGP |
| > non-ACP_69 |
| LLAAGFCPAVLCH |
| > non-ACP_70 |
| AASVNDEQHQRIIKYGRALVLDIVEQ |
| > non-ACP_71 |
| IARLREDGIQKRVIQEGRGELPDFQDG |
| > non-ACP_72 |
| FIVVMNILALTLPFLAAEVQN |
| > non-ACP_73 |
| CQNGRRANRTVRFARTA |
| > non-ACP_74 |
| WVTVRSQQRGLFPAI |
| > non-ACP_75 |
| LLRSCPLQGSPGRPRSV |
| > non-ACP_76 |
| LNDGHFMPVLGFGTYAPPEVPRNRAVEV |
| > non-ACP_77 |
| HLGRPSAPTIVAQPVSGLASPASFQPEQFQYTLDNNVLT |
| > non-ACP_78 |
| RPEPGGCCCRRTVRANGC |
| > non-ACP_79 |
| SWVEENRASFQPPVCNKLMHR |
| > non-ACP_80 |
| VFHRVRWAPELGASLG |
| > non-ACP_81 |
| RAKVLTLDGMNPRVRRVEYAVRGPIVQRALELEQELRQ |
| > non-ACP_82 |
| LLQRARLAEQAERYDDMASAMKAVTELNEPLS |
| > non-ACP_83 |
| ALIQKLNSDPQFVLAQNVGTTHDLLDICLKRATVQRA |
| > non-ACP_84 |
| AALPMLWTGLVLLGLLGFPQTPAQGHDTVQPNFQQ |
| > non-ACP_85 |
| QRRQNDSSVFLAIMVAAAVES |
| > non-ACP_86 |
| CNAPGCGQRFTNEDHLAVHKHKHEMTLKFGPARTDS |
| > non-ACP_87 |
| LVLLTVQNSALILTLNYSRIMPGYD |
| > non-ACP_88 |
| TVLSPPQRFKRILQAMMLAVAVV |
| > non-ACP_89 |
| ISRGLLLLLAALCCLAPSFL |
| > non-ACP_90 |
| VAGTESAQGPPGPAASLELWLNKATDPS |

| |
|---|
| > non-ACP_91 |
| QYLRIRTVQPEPDYGAAV |
| > non-ACP_92 |
| ASPTQTPPTTSTIRVARRSRVALVAM |
| > non-ACP_93 |
| TIWRNQHTYKMATSASANLSKIVKKNYMELPQDGKVQ |
| > non-ACP_94 |
| LSITRGLLLLAALCCLAPIS |
| > non-ACP_95 |
| ILLSIWRQSSGRGKLPPGPIPLPIIGNIFQ |
| > non-ACP_96 |
| LLLLSWVALGPRSLEGADPGTPGEAEGPACP |
| > non-ACP_97 |
| LRVKRAMQEASFMPPLLPPAAHQRFSTVPAVP |
| > non-ACP_98 |
| GLLLLAGLCCLVFGIMAEDAQVAQGPSQQI |
| > non-ACP_99 |
| RHVGLLCATGPQRWRF |
| > non-ACP_100 |
| AAVALARPKPPLRHQEHLQNEPDS |
| > non-ACP_101 |
| SRVNDQSQASRNGLKGKVLTLDTMNPCV |
| > non-ACP_102 |
| AAALGPGVLQATRAFHTGQPRLAPLPPLPEYGGK |
| > non-ACP_103 |
| LCTSGLWTAQASTNESSNSHRGLAPTNV |
| > non-ACP_104 |
| PAIQPVLSGLSRIVNGEEA |
| > non-ACP_105 |
| GCQASLSTAQERLGHPGVPTREGVR |
| > non-ACP_106 |
| RKVLILTLVVAACGFVLWSSNGR |
| > non-ACP_107 |
| GRVRSRCPGPALLLLLALAARPALAGPPAAALQ |
| > non-ACP_108 |
| CRITKPALLVLNQETAKVVQT |
| > non-ACP_109 |
| KAEVCMAVPWLSLQ |
| > non-ACP_110 |
| SHLELNNGTKMPTLGLGT |
| > non-ACP_111 |
| LLLPEAAAERDAREKLALWDRRPDTTAPL |
| > non-ACP_112 |
| LLALSLVLLYRYATYSHGFFKKLGIPGPKPLPLFGNVLS |
| > non-ACP_113 |
| LLSLWRQSSGRGKLPPGPTPLPVIGNILQIGIKD |
| > non-ACP_114 |

| |
|---|
| AFKSMEVANFYYEADCLAAAYGGKAAPAAPPADRPGPR |
| > non-ACP_115 |
| SLFWAARPLQRCGQLVRMAIRAQH |
| > non-ACP_116 |
| MRPPRTLTSTATMSALSTSMPMEIDDVMDEDAVNGQA |
| > non-ACP_117 |
| LLSLIGFCWAQYDP |
| > non-ACP_118 |
| LWARSKNDQLRISFPPGLCWG |
| > non-ACP_119 |
| PQGFDVDRDAKKLNKACKGMGTNEAAIIEILSG |
| > non-ACP_120 |
| IFCLILWVVKAWQPRLPKGLKSPPGPWGWPLLG |
| > non-ACP_121 |
| LDAASPGPLALLGLLFAATLLLSALFLL |
| > non-ACP_122 |
| VTLLFKLYCLA |
| > non-ACP_123 |
| ATRAAAARLVGTAASRTPAAARH |
| > non-ACP_124 |
| RNKLDLETLTDILEHQIR |
| > non-ACP_125 |
| RRLVLQARTYAQAAASPAPAAGPGQMSFTFASPTQVFF |
| > non-ACP_126 |
| PGRSRSAADDINPAPANM |
| > non-ACP_127 |
| LLSALTLETWVLLAVILVLLYRLG |
| > non-ACP_128 |
| VMGHGLCPQGARAKAAIPAALRDHEST |
| > non-ACP_129 |
| FLVSIAGLLYALVQLGQPCDCLPPLRAAA |
| > non-ACP_130 |
| VRSVRAAVGGLRAISAPSAPCLPRPWGLRAG |
| > non-ACP_131 |
| RGGCWPRGLQQLLVPGG |
| > non-ACP_132 |
| APATPPRPLKRKKLQFTDVTPESSP |
| > non-ACP_133 |
| EQAERYDDMAAAMKAVTEQGHELSNEERNL |
| > non-ACP_134 |
| MPNDPSDNQLK |
| > non-ACP_135 |
| TELLLAITVFCLGFWVVRALRTQVP |
| > non-ACP_136 |
| LTADLLGAPFFTLPKELQLALLERQTVFL |
| > non-ACP_137 |
| GHGRLVEIQGRLGVRIER |

| |
|---|
| > non-ACP_138 |
| LPFKLLLFVLLDGWTRLTH |
| > non-ACP_139 |
| QALWLVLVLSMPPVLVAAVVGTLVSLVQ |
| > non-ACP_140 |
| TASWLGNELHSFAEMTMMKIQGIR |
| > non-ACP_141 |
| KHRTVLFRRWMAIICCLI |
| > non-ACP_142 |
| LHRAQDYRRELDTLQSLLTTSQSSELQAAAALLKC |
| > non-ACP_143 |
| TKGNDIADLDAVAQTLKKPADDANKAVN |
| > non-ACP_144 |
| QLEMALAKLKKDMMRGGDAKQYQVWQS |
| > non-ACP_145 |
| VSVDNPNDLMLMQWSLIRITIQEE |
| > non-ACP_146 |
| QGKAGEVIVTGLRTEQLSLSDAKLLLEAAMR |
| > non-ACP_147 |
| RGVGGPSQQVMTPQGRGTVAAAAAAATASIAGAP |
| > non-ACP_148 |
| VGKSSKMLQHIDYRMRCILQDGRIFIGTFKA |
| > non-ACP_149 |
| HHKDSSIHHLRLSANDAEDSLRM |
| > non-ACP_150 |
| LNPDPCKPLAFI |
| > non-ACP_151 |
| SLINQQITQVGHGGQAGRLTETNPLTENS |
| > non-ACP_152 |
| IACLLPRVSNLELKQGKAGEVIVTGLR |
| > non-ACP_153 |
| EPSTGLMLVEESRWVHLGRELLPATAP |
| > non-ACP_154 |
| NATSSQDQFAKWARLNRKYEQLSQ |
| > non-ACP_155 |
| EPQKTIWRLAIFLHLPLRLAVAKIY |
| > non-ACP_156 |
| LFLVNVLAFGLAGYCFVRHNARCEAGV |
| > non-ACP_157 |
| PTYERLNDPKNVFFRMPFARL |
| > non-ACP_158 |
| WNHYLSCASPCPGYRLLCRIN |
| > non-ACP_159 |
| EAAMAAAAAPGSGNGVGGGGGTAG |
| > non-ACP_160 |
| LGSGHHDGSGDSLEMSSLDRAQ |
| > non-ACP_161 |

| |
|---|
| FYQNRAAAFEQLQKWKEVAQDCT |
| > non-ACP_162 |
| ELNPKYVKALFRRAKAHE |
| > non-ACP_163 |
| MAEALLLRATFYLLIGSANAAKPD |
| > non-ACP_164 |
| RANALIKRGTMCMQQQQPMLSTQ |
| > non-ACP_165 |
| RPKFALAQAQKCFALYRQAYTANNSSQVQAAMKGFEE |
| > non-ACP_166 |
| YAQALTDQQQFGKADEMYDKCI |
| > non-ACP_167 |
| GNMEKAIDMFNKAINLAKSEMEMAHLYSLC |
| > non-ACP_168 |
| LSVALGPRSRSYRAPPPPRRRPGPHS |
| > non-ACP_169 |
| MQESLRLQQQALEAR |
| > non-ACP_170 |
| RKRLKEERQRQKKEARIAA |
| > non-ACP_171 |
| GETRPSECIAALFDDRARPP |
| > non-ACP_172 |
| LLEFAQLHNISSRTLQRQFVAS |
| > non-ACP_173 |
| AASVNPLAAVELQRLSAQER |
| > non-ACP_174 |
| QNCVFADNIKVGQMTEPLKDQQIIL |
| > non-ACP_175 |
| LTMMAPAVAQQTDDETFV |
| > non-ACP_176 |
| QQIQGGKELKDALAQL |
| > non-ACP_177 |
| ERFTSNQMFFDLAQASASGGLNNKKI |
| > non-ACP_178 |
| RQQEFLLLNGWLQLQCGHAERACILLD |
| > non-ACP_179 |
| NWLCLSRAQQLNGDLDKARHAYQ |
| > non-ACP_180 |
| YKELYYRLLTSPAADAGNTL |
| > non-ACP_181 |
| EWRDLKGRADINKDDVV |
| > non-ACP_182 |
| GWVTTLGPGSRGYRAPPPPRRSRE |
| > non-ACP_183 |
| QMIENWRRQQQARREKAQADKERRARLQ |
| > non-ACP_184 |
| QRKKKEARAAAMAAAAAQDPADSETP |

| |
|---|
| > non-ACP_185 |
| APATRSRYLLRLTVTLGPRSRSYHAPPPPRRRP |
| > non-ACP_186 |
| ENWRKQKRERWEKIQADKERRARLQAEAQE |
| > non-ACP_187 |
| LKEERQRQKKEARIAAMASAEGQDSAEAQD |
| > non-ACP_188 |
| TLDRDGTLVRLRFTMVALITVCCPLVAFFF |
| > non-ACP_189 |
| LFLTGCKVDLYTGISQKEGNEMLALL |
| > non-ACP_190 |
| LDRDGTLVRLRFTLVALVTVCCPLVAFLFCVL |
| > non-ACP_191 |
| LWRLCIGLHSAPRFLVAMAYLKYYQGTPC |
| > non-ACP_192 |
| VAGTTAVAGQVPIMGAVKFGGDVCAAG |
| > non-ACP_193 |
| AKLRRKHDKAMDEYEAMNKKLTAQK |
| > non-ACP_194 |
| QLRSRSASPASTAKAQKAQ |
| > non-ACP_195 |
| LIWTIFILHIAIFLVNTIGAATIDNLLWLLYLKLP |
| > non-ACP_196 |
| PDPPADSPLDQTIQHLQGLTIQELPDPPTHLPE |
| > non-ACP_197 |
| ERFAQLCEEHGILRENIIDLSNANRCL |
| > non-ACP_198 |
| EEGLQGKLRLLRLLHQTNPYPQGPGTASQRRNRRRRRRR |
| > non-ACP_199 |
| PTDSPLDRAIQHLQRLTIQELPDPPTDLPESNSNQ |
| > non-ACP_200 |
| GPGTANQRRQRRRRWRRRWQQLLALA |
| > non-ACP_201 |
| EEELRKRLRLIHLLHQTIDSYPTGPGTANQRRQRR |
| > non-ACP_202 |
| TDTPLDLAIQQLQNLAIESIPDPPTNTPEALCD |
| > non-ACP_203 |
| LRLIHFLHQTTDPYPQGPGTANQRRRR |
| > non-ACP_204 |
| PVDTPLDLAIQQLQGLAIEELPDPPTSAPEPLNDV |
| > non-ACP_205 |
| NTNVTPHLLAGMRLIAVQQPEDPLRVL |
| > non-ACP_206 |
| QTLVHNGGRLPPDLQLSAEDSSSTPS |

**Supporting Information S2: The Independent dataset contains 150 anticancer peptides and 150 non-anticancer peptides (see Eq.2)**

**I. 150 anticancer peptides**

| |
|---|
| > ACP_1 |
| AAKKWAKAKWAKAKKWAKAA |
| > ACP_2 |
| AAVPIVNLKDELLFPSWEALFSGSE |
| > ACP_3 |
| AAWKWAWAKKWAKAKKWAKAA |
| > ACP_4 |
| AIGSILGALAKGLPTLISWIKNR |
| > ACP_5 |
| AKRHHGYKRKFH |
| > ACP_6 |
| ALSKALSKALSKALSKALSKALSK |
| > ACP_7 |
| ALWKDILKNVGKAAGKAVLNTVTDMVNQ |
| > ACP_8 |
| ALWKTMLKKLGTMALHAGKAALGAAADTISQGTQ |
| > ACP_9 |
| AWKKWAKAWKWAKAKWWAKAA |
| > ACP_10 |
| FAFAKIIAKIAKKII |
| > ACP_11 |
| FAFGKGIGKIGKKGL |
| > ACP_12 |
| FAKAIAKIAFGKGIGKVGKKLL |
| > ACP_13 |
| FAKALAKLAKKLL |
| > ACP_14 |
| FAKFLAKFLKKAL |
| > ACP_15 |
| FAKIIAKIAKIAKKIL |
| > ACP_16 |
| FAKKFAKKFKKFAKKFAKFAFAF |
| > ACP_17 |
| FAKKLAKKAKLAKKL |
| > ACP_18 |
| FAKKLAKKLKKLAKKLAKKWKL |
| > ACP_19 |
| FAKKLAKKLKKLAKKLAKLALALKALALKAL |
| > ACP_20 |
| FAKKLAKKLKKLAKKLIGAVLKV |
| > ACP_21 |
| FAKKLAKLAKKALAL |
| > ACP_22 |

| |
|---|
| FAKKLAKLAKKLAKAL |
| > ACP_23 |
| FAKKLAKLAKKLAKLALAL |
| > ACP_24 |
| FAKKLAKLAKKLLAL |
| > ACP_25 |
| FAKKLAKLALKLAKL |
| > ACP_26 |
| FAKKLLAKALKL |
| > ACP_27 |
| FAKLAKKALAKLL |
| > ACP_28 |
| FAKLFAKLAKKFAL |
| > ACP_29 |
| FAKLLAKAFKKAL |
| > ACP_30 |
| FAKLLAKALKKAL |
| > ACP_31 |
| FAKLLAKALKKFAL |
| > ACP_32 |
| FAKLLAKALKLKL |
| > ACP_33 |
| FAKLLAKFLKKAL |
| > ACP_34 |
| FAKLLAKLAKAKA |
| > ACP_35 |
| FAKLLAKLAKKAL |
| > ACP_36 |
| FAKLLAKLAKKFAL |
| > ACP_37 |
| FAKLLFKALKKAL |
| > ACP_38 |
| FAKLLKLAAKKLL |
| > ACP_39 |
| FAKLWAKLAFGKGIGKVGKKLL |
| > ACP_40 |
| FAKLWAKLAKKL |
| > ACP_41 |
| FALAAKALKKLAKKLKKLAKKAL |
| > ACP_42 |
| FALAKALKKAL |
| > ACP_43 |
| FALAKKALKKAKKAL |
| > ACP_44 |
| FALAKLAKKAKAKLKKALKAL |
| > ACP_45 |

| |
|---|
| FALALKALKKALKKLKKALKKAL |
| > ACP_46 |
| FALALKALKKLLKKLKKLAKKAL |
| > ACP_47 |
| FALALKLAKKAL |
| > ACP_48 |
| FALKALKKLKKALKKAL |
| > ACP_49 |
| FALLKALKKAL |
| > ACP_50 |
| FALLKALLKKAL |
| > ACP_51 |
| FKLAFKLAKKAFL |
| > ACP_52 |
| FKRLAKIKVLRLAKIKR |
| > ACP_53 |
| FLGALFHALSKLL |
| > ACP_54 |
| FLGALFKALSHLL |
| > ACP_55 |
| FLGALFKALSKLL |
| > ACP_56 |
| FLGMIPGLIGGLISAFK |
| > ACP_57 |
| FLGMIPKLIKKLIKAFK |
| > ACP_58 |
| FLGWLFKWAKK |
| > ACP_59 |
| FLGWLFKWAWK |
| > ACP_60 |
| FLKLLKKLAAKFLPTIICKISYKC |
| > ACP_61 |
| FLKLLKKLAAKLF |
| > ACP_62 |
| FLPLIGRVLSGIL |
| > ACP_63 |
| FLSLIPKLVKKIIKAFK |
| > ACP_64 |
| FLSLIPSLVGGSISAFK |
| > ACP_65 |
| FLWWLFKWAWK |
| > ACP_66 |
| GFFALIPKIISSPLFKTLLSAVGSALSSSGGQE |
| > ACP_67 |
| GFKMALKLLKKVL |
| > ACP_68 |
| GIGKFLHAAKKFAKAFVAEIMNS |

| |
|---|
| > ACP_69 |
| GIGKFLHSAKKWGKAFVGQIMNC |
| > ACP_70 |
| GIGKFLKKAKKFAKAFVKIINN |
| > ACP_71 |
| GKFMSLLKHILK |
| > ACP_72 |
| GKWKKILGHLIR |
| > ACP_73 |
| GKWKKILGKLIR |
| > ACP_74 |
| GKWKSLLKHILK |
| > ACP_75 |
| GKWLSLLKHILK |
| > ACP_76 |
| GKWMKLLKHILK |
| > ACP_77 |
| GKWMSFLKHILK |
| > ACP_78 |
| GKWMSLLKHWLK |
| > ACP_79 |
| GKWMSLLKKILK |
| > ACP_80 |
| GKWMSLWKHILK |
| > ACP_81 |
| GKWMTLLKHILK |
| > ACP_82 |
| GKWSKILGKLIR |
| > ACP_83 |
| GLFAVIKKVAAVIKKL |
| > ACP_84 |
| GLFAVIKKVAAVIRRL |
| > ACP_85 |
| GLFAVIKKVASVIKGL |
| > ACP_86 |
| GLFDVIAKVASVIKKL |
| > ACP_87 |
| GLFDVIKAVASVIGGL |
| > ACP_88 |
| GLFDVIKKVAAVIGGL |
| > ACP_89 |
| GLFDVIKKVASVIKKL |
| > ACP_90 |
| GLFGKLIKKFARKAISYAVKKARGKH |
| > ACP_91 |
| GLFSVLGAVAKHVLPHVVPVIAEK |
| > ACP_92 |

| |
|---|
| GMWSKILKHLIR |
| > ACP_93 |
| GMWSKLLGHLLR |
| > ACP_94 |
| GQVWEATATVNAIRGSVTPAVSQFNARTAD |
| > ACP_95 |
| GRRKRKWLRRIGKGVKIIGGAALDHL |
| > ACP_96 |
| GSEGPLKPGARIFSFDGKDVLRHPT |
| > ACP_97 |
| GVGSPYVSRLLGICL |
| > ACP_98 |
| GWRTLLKKAEVKTVGKLALKHYL |
| > ACP_99 |
| HHPHGHHPHGHHPHGHHPHG |
| > ACP_100 |
| HPTWPQKSVWHGSDPNGRRLTESY |
| > ACP_101 |
| HSHRDFQPVLHLVALNSPLSGGMRG |
| > ACP_102 |
| ILPWKWPWWPWRR |
| > ACP_103 |
| ILRWPWWPWRRK |
| > ACP_104 |
| KAAKKAAKAAKKAAKAAKKAA |
| > ACP_105 |
| KIAKVALAKLGIGAVLKVLTTGL |
| > ACP_106 |
| KILRGVAKKILRTFLRRISKDILTGKK |
| > ACP_107 |
| KILRGVAKKIMRTFLRRISKKILTGKK |
| > ACP_108 |
| KISKRILTGKK |
| > ACP_109 |
| KKKFPWWWPFKKKCKKKFPWWWPFKKKC |
| > ACP_110 |
| KLAKKLAKLAKLAKAL |
| > ACP_111 |
| KNWKKILGKIIKVVK |
| > ACP_112 |
| KNWKKILKKIIKVVK |
| > ACP_113 |
| KTKLFKKFAKKLAKKLKKLAKKL |
| > ACP_114 |
| KWFKKIPKFLHLLKKF |
| > ACP_115 |
| KWKFKKIPKFLHLAKKF |

| |
|---|
| > ACP_116 |
| KWKKFLKIGIGKFLHLAKKF |
| > ACP_117 |
| KWKKLLKKPPPLLKKLLKKL |
| > ACP_118 |
| KWKLAKKALALL |
| > ACP_119 |
| KWKLFAKIGIGKFLHLAKKF |
| > ACP_120 |
| KWKLFKKALKKLKKALKKAL |
| > ACP_121 |
| KWKLFKKIGIGAFLHLAKKF |
| > ACP_122 |
| KWKLFKKIGIGAVLKVLKKG |
| > ACP_123 |
| KWKLFKKIGIGKFKLAKKF |
| > ACP_124 |
| KWKLFKKIGIGKFLHSAKKF |
| > ACP_125 |
| KWKLFKKIPHLAKKF |
| > ACP_126 |
| KWKLFKKIPLAKKF |
| > ACP_127 |
| KWKLFKKIPLKKF |
| > ACP_128 |
| KWKLFKKKTKLFKKFAKKLAKKL |
| > ACP_129 |
| KWKSFAKTFKSAKKTVAHTALKAISS |
| > ACP_130 |
| KWKSFLKTFKSAKKTVAHTAAKAISS |
| > ACP_131 |
| KYKKALKKLAKLL |
| > ACP_132 |
| LGGIVSAVKKIVDFLG |
| > ACP_133 |
| LLGDFKRIVQRIKDF |
| > ACP_134 |
| LPKWKVFKKIEKVGRNIRNGIVKAGPAIAVLGEAKALG |
| > ACP_135 |
| MNFNKLFVFVALVLAVCIGQSEAGWLKKIGKKIERVGQHTRDATIQTIGVAQ |
| > ACP_136 |
| MNFQQRLQSLWTLARPFCPPLLATASQMQMVVLPCLGFTLLLWSQVSGAQGQEFHFGPCQVKGVVPQ |
| > ACP_137 |
| MPKEKVFLKIEKMGRNIRN |
| > ACP_138 |
| MPRWRLFRRIDRVGKQIKQGILRAGPAIALVGDARAVG |
| > ACP_139 |

| |
|---|
| MRKWFHNVLSSGQLLADKWPAWDYNRK |
| > ACP_140 |
| MWKEFHNVLSSGQLLADKRWARWYNRW |
| > ACP_141 |
| MWKWFHNVLSSGQLLADKWWAWWYNWW |
| > ACP_142 |
| MWKWFHNVLSSWQLLADKRPARDYNRK |
| > ACP_143 |
| NKWKKILGKIIKVVK |
| > ACP_144 |
| NYHNRTVEVRTLKSFSTLANNFVLIVSQLQPSQENEMFSIRGDSAHRRFLLFRRAFKQLDVEAAL |
| > ACP_145 |
| PDEDAINDALNKVCSTGRRQRSICKQLLKK |
| > ACP_146 |
| PEWFKCRRWQWRMKKLGA |
| > ACP_147 |
| RGDLLRHVVKILEKYL |
| > ACP_148 |
| RKAFRWAWRMLKKAAPSITCVR |
| > ACP_149 |
| RWGKWFKKATHVGKHVGKAALTAYL |
| > ACP_150 |
| SWLSKTAKKLENSAKKRISEGIAIAIQGGPR |
| **II. 150 non-anticancer peptides** |
| > non-ACP_1 |
| AAEFPDFYDSEEQMGPHQEAEDEKDRADQRVLTEEEKKELENLAAMDLELQKIAEKFSQR |
| > non-ACP_2 |
| AAKPMGITCDLLSLWKVGHAACAAHCLVLGDVGGYCTKEGLCVCKE |
| > non-ACP_3 |
| ACNFQSCWATCQAQHSIYFRRAFCDRSQCKCVFVRG |
| > non-ACP_4 |
| ACYCRIGACVSGERLTGACGLNGRIYRLCCR |
| > non-ACP_5 |
| AEVAPAPAAAAPAKAPKKKAAAKPKKAGPS |
| > non-ACP_6 |
| AFPPPNVPGPRFPPPNFPGPRFPPPNFPGPRFPPPNFPGPRFPPPNFPGPPFPPPIFPGPWFPPPPPFRPPPFGPPRFP |
| > non-ACP_7 |
| AGANDLCQECEDIVHLLTKMTKEDAFQDTIRKFLEQECDILPLKLLVPRCRQVLDVYLPLVIDYFQGQIKPKA<br>ICSHVGLC |
| > non-ACP_8 |
| AGDPLADPNSQIVRQIMSNAAWGPPLVPERFRGMAVGAAGGVTQTVLQGAAAHMPVNVPIPKVPMGPSWNGSKG |
| > non-ACP_9 |
| ALPKKLKYLNLFNDGFNYMGVV |
| > non-ACP_10 |
| ALSILKGLEKLAKMGIALTNCKATKKC |
| > non-ACP_11 |
| ALWKDILKNAGKAALNEINQLVNQ |
| > non-ACP_12 |

| |
|---|
| ANTAFVSSAHNTQKIPAGAPFNRNLRAMLADLRQNAAFAG |
| > non-ACP_13 |
| APPGARPPPGPPPPGPPPPGP |
| > non-ACP_14 |
| AQRCGDQARGAKCPNCLCCGKYGFCGSGDAYCGAGSCQSQCRGCR |
| > non-ACP_15 |
| ATAVDFGPHGLLPIRPIRIRPLCGKDKS |
| > non-ACP_16 |
| ATCDLFSFRSKWVTPNHAACAAHCLLRGNRGGRCKGTICHCRK |
| > non-ACP_17 |
| ATCDLLSGIGVQHSACALHCVFRGNRGGYCTGKGICVCRN |
| > non-ACP_18 |
| ATCDLLSGTGINHSACAAHCLLRGNRGGYCNGKAVCVCRN |
| > non-ACP_19 |
| ATCDLLSMWNVNHSACAAHCLLLGKSGGRCNDDAVCVCRK |
| > non-ACP_20 |
| ATCYCRTGRCATRESLSGVCEISGRLYRLCCR |
| > non-ACP_21 |
| ATPATPTVAQFVIQGSTICLVC |
| > non-ACP_22 |
| ATRVVYCNRRSGSVVGGDDTVYYEG |
| > non-ACP_23 |
| AVDFSSCARMDVPGLSKVAQGLCISSCKFQNCGTGHCEKRGGRPTCVCDRCGRGGGEWPSVPMPKGRSSRGRRHS |
| > non-ACP_24 |
| AVTCNTVVSSLAPCVPFFAGSAAQPTAACCNGVRSLNSAARTTPDRRTACNCIKSSASSIGLNYNKAAKLPSRCTVNVTVPISPSVNCAT |
| > non-ACP_25 |
| CAWYNISCRLGNKGAYCTLTVECMPSCN |
| > non-ACP_26 |
| CLAGRLDKQCTCRRSQPSRRSGHEVGRPSPHCGPSRQCGCHMD |
| > non-ACP_27 |
| CSCRTSSCRFGERLSGACRLNGRIYRLCC |
| > non-ACP_28 |
| DAPGHPGKHYLQVNVPSDVRTIGVAGGGVQQCFRVTPGAWNDTRALVSNGAQVEVWGYTVADCANRTTANQKYYDKAAAPSDSSTYFWFTLKNLRV |
| > non-ACP_29 |
| DDMTMKPTPPPQYPLNLQGGGGGGSGDGFGFAVQGHQKVWTSDNGRHEIGLNGGYGQHLGGPYGNSEPSWKVGSTYTYRFPNF |
| > non-ACP_30 |
| DFKDWMKTAGEWLKKKGPGILKAAMAAAT |
| > non-ACP_31 |
| DHHHDHGHDDHEHEELTLEKIKEKIKDYADKTPVDQLTERVQAGRDYLLGKGARPSHLPARVDRHLSKLTAAEKQELADYLLTFLH |
| > non-ACP_32 |
| DIQIPGIKKPTHRDIIIPNWNPNVRTQPWQRFGGNKS |
| > non-ACP_33 |
| DLRFLYPRGKLPVPTLPPFNPKPIYIDMGNRY |

| |
|---|
| > non-ACP_34 |
| DLRFWNPREKLPLPTLPPFNPKPIYIDMGNRY |
| > non-ACP_35 |
| DSHEKRHHGYRRKFHEKHHSHREFPFYGDYGSNYLYDN |
| > non-ACP_36 |
| DVQCGEGHFCHDQTCCRASQGGACCPYSQGVCCADQRHCCPVGF |
| > non-ACP_37 |
| EADEPLWLYKGDNIERAPTTADHPILPSIIDDVKLDPNRRYA |
| > non-ACP_38 |
| EIRLPEPFRFPSPTVPKPIDIDPILPHPWSPRQTYPIIARRS |
| > non-ACP_39 |
| EKKCPGRCTLKCGKHERPTLPYNCGKYICCVPVKVK |
| > non-ACP_40 |
| ELPKLPDDKVLIRSRSNCPKGKVWNGFDCKSPFAFS |
| > non-ACP_41 |
| ENDHRMPNNLNRPNNLSKGGAKCGAAIAGGLFGIPKGPLAWAAGLANVYSKCN |
| > non-ACP_42 |
| ENFFKEIERAGQRIRDAIISAAPAVETLAQAQKIIKGGD |
| > non-ACP_43 |
| ERGSRGQRCGPGEVFNQCGSACPRVCGRPPAQACTLQCVSGCFCRRGYIRTQRGGCIPERQCHQR |
| > non-ACP_44 |
| ETESTPDYLKNIQQQLEEYTKNFNTQVQNAFDSDKIKSEVNNFIESLGKILNTEKKEAPK |
| > non-ACP_45 |
| FAEPLPSEEEGESYSKEPPEMEKRYGGFM |
| > non-ACP_46 |
| FFRHLFRGAKAIFRGARQGWRAHKVVSRYRNRDVPETDNNQEEP |
| > non-ACP_47 |
| FFSASCVPGADKGQFPNLCRLCAGTGENKCA |
| > non-ACP_48 |
| FGLPMLSILPKALCILLKRKC |
| > non-ACP_49 |
| FSFKRLKGFAKKLWNSKLARKIRTKGLKYVKNFAKDMLSEGEEAPPAAEPPVEAPQ |
| > non-ACP_50 |
| FSKYERQKDKRPYSERKNQYTGPQFLYPPERIPPQKVIKWNEEGLPIYEIPGEGGHAEPAAA |
| > non-ACP_51 |
| GEILCNLCTGLINTLENLLTTKGADKVKDYISSLCNKASGFIATLCTKVLDFGIDKLIQLIEDKVDANAICAKIHAC |
| > non-ACP_52 |
| GFGCPLDQMQCHRHCQTITGRSGGYCSGPLKLTCTCYR |
| > non-ACP_53 |
| GFKLKGMARISCLPNGQWSNFPPKCIRECAMVSS |
| > non-ACP_54 |
| GGLKKLGKKLEGVGKRVFKASEKALPVAVGIKALGK |
| > non-ACP_55 |
| GGLKKLGKKLEGVGKRVFKASEKALPVLTGYKAIG |
| > non-ACP_56 |
| GILDSFKQFAKGVGKDLIKGAAQGVLSTMSCKLAKTC |
| > non-ACP_57 |

| |
|---|
| GILDSLKNLAKNAGQILLNKASCKLSGQC |
| > non-ACP_58 |
| GILDTLKNLAISAAKGAAQGLVNKASCKLSGQC |
| > non-ACP_59 |
| GILDTLKQFAKGVGKDLVKGAAQGVLSTVSCKLAKTC |
| > non-ACP_60 |
| GKQYFPKVGGRLSGKAPLAAKTHRRLKP |
| > non-ACP_61 |
| GKWGWIYITILFADVGGFKSSRHPEERRVQERRFKRITRGPD |
| > non-ACP_62 |
| GLFRRLRDSIRRGQQKILEKARRIGERIKDIFRG |
| > non-ACP_63 |
| GLFSILRGAAKFASKGLGKDLTKLGVDLVACKISKQC |
| > non-ACP_64 |
| GLFTLIKCAYQLIAPTVACN |
| > non-ACP_65 |
| GLGSVFGRLARILGRVIPKVAKKLGPKVAKVLPKVMKEAIPMAVEMAKSQEEQQPQ |
| > non-ACP_66 |
| GLLCYCRKGHCKRGERVRGTCGIRFLYCCPRR |
| > non-ACP_67 |
| GLLDSIKGMAISAGKGALQNLLKVASCKLDKTC |
| > non-ACP_68 |
| GLLDSLKNLAINAAKGAGQSVLNTLSCKLSKTC |
| > non-ACP_69 |
| GLLNGLALRLGKRALKKIIKRLCR |
| > non-ACP_70 |
| GLLRASSVWGRKYYVDLAGCAKA |
| > non-ACP_71 |
| GLLSGVLGVGKKVDCGLSGLC |
| > non-ACP_72 |
| GLLSRLRDFLSDRGRRLGEKIERIGQKIKDLSEFFQS |
| > non-ACP_73 |
| GLMSVLGHAVGNVLGGLFKS |
| > non-ACP_74 |
| GLRSKIWLWVLLMIWQESNKFKKM |
| > non-ACP_75 |
| GLVSSIGKALGGLLADVVKTKEQPA |
| > non-ACP_76 |
| GLVSSIGRALGGLLADVVKSKEQPA |
| > non-ACP_77 |
| GPDSCNHDRGLCRVGNCNPGEYLAKYCFEPVILCCKPLSPTPTKT |
| > non-ACP_78 |
| GRPNPVNNKPTPHPRL |
| > non-ACP_79 |
| GRPNPVNTKPTPYPRL |
| > non-ACP_80 |
| GRRRRSVQWCAVSQPEATKCFQWQRNMRKVRGPPVSCIKRDSPIQCIQA |

| |
|---|
| > non-ACP_81 |
| GSCSCSGTISPYGLRTCRATKTKPSHPTTKETHPQTLPT |
| > non-ACP_82 |
| GSKKPVPIIYCNRRTGKCQRM |
| > non-ACP_83 |
| GSNFCDSKCKLRCSKAGLADRCLKYCGICCEECKCVPSGTYGNKHECPCYRDKKNSKGKSKCP |
| > non-ACP_84 |
| GSQLVYREWVGHSNVIKGPP |
| > non-ACP_85 |
| GSVLNCGETCLLGTCYTTGCTCNKYRVCTKD |
| > non-ACP_86 |
| GTCKAECPTWEGICINKAPCVKCCKAQPEKFTDGHCSKILRRCLCTKPC |
| > non-ACP_87 |
| GTPGFQTPDARVISRFGFN |
| > non-ACP_88 |
| GTWDDIGQGIGRVAYWVGKALGNLSDVNQASRINRKKKH |
| > non-ACP_89 |
| GVFLDALKKFAKGGMNAVLNPK |
| > non-ACP_90 |
| GVFSFLKTGAKLLGSTLLKMAGKAGAEHLACKATNQC |
| > non-ACP_91 |
| GVIKSVLKGVAKTVALGML |
| > non-ACP_92 |
| GVITDALKGAAKTVAAELLRKAHCKLTNSC |
| > non-ACP_93 |
| GVLDILKGAGKDLLAHALSKISEKV |
| > non-ACP_94 |
| GVLDILTGAGKDLLAHALSKLSEKV |
| > non-ACP_95 |
| GVVDILKGAGKDLLAHLVGKISEKV |
| > non-ACP_96 |
| GWANTLKNVAGGLCKITGAA |
| > non-ACP_97 |
| HHQELCTKGDDALVTELECIRLRISPETNAAFDNAVQQLNCLNRACAYRKMCATNNLEQAMSVY FTNEQIKEIHDAATACDPEAHHEHDH |
| > non-ACP_98 |
| HNKQEGRDHDKSKGHFHRVVIHHKGGKAH |
| > non-ACP_99 |
| HRHQGPIFDTRPSPFNPNQPRPGPIY |
| > non-ACP_100 |
| HVDKKVADKVLLLKQLRIMRLLTRL |
| > non-ACP_101 |
| IFGAILPLALGALKNLIK |
| > non-ACP_102 |
| IIEKLVNTALGLLSGL |
| > non-ACP_103 |
| IIGLVSKGTCVLVKTVCKKVLKQG |

| |
|---|
| > non-ACP_104 |
| IKITTMLAKLGKVLAHV |
| > non-ACP_105 |
| ILENLLARSTNEDREGSIFDTGPIRRPKPRPRPRPEG |
| > non-ACP_106 |
| ITCQQVTSELGPCVPYLTGQGIP |
| > non-ACP_107 |
| IYWIADQFGIHLATGTARKLLDAMASGASLGTAFAAILGVTLPAWALAAAGALGATAA |
| > non-ACP_108 |
| KCKWWNISCDLGNNGHVCTLSHECQVSCN |
| > non-ACP_109 |
| KLWEAFWAVKDTMQAQDNITSARLLQQEVLQNVSDAESCYLVHTLLEFYLKTVFKNYHNRTVE |
| > non-ACP_110 |
| KQEGRDHDKSKGHFHMIVIHHKGGQAHHG |
| > non-ACP_111 |
| KQQLATEAESAGPIL |
| > non-ACP_112 |
| KRFGRLAKSFLRMRILLPRRKILLAS |
| > non-ACP_113 |
| KRFHSVGSLIQRHQQMIRDKSEATRHGIRIITRPKLLLAS |
| > non-ACP_114 |
| KRRGSVTTRYQFLMIHLLRPKKLFA |
| > non-ACP_115 |
| KRRHWFPLSFQEFLEQLRRFRDQLPFP |
| > non-ACP_116 |
| KSCCRSTQARNIYNAPRFAGGSRPLCALGSGCKIVDDKKTPPND |
| > non-ACP_117 |
| LCLDQKPEMEPFRKDAQQALEPSRQRRWLHRRCLSGRGFCRAICSIFEEPVRGNIDCYFGYNCCRRMFSHYRTS |
| > non-ACP_118 |
| LIDHLGAPRWAVDTILGAIAVGNLASWVLALVPGPGWAVKAGLATAAAIVKHQGKAAAAAW |
| > non-ACP_119 |
| LLGRCKVKSNRFHGPCLTDTHCSTVCRGEGYKGGDCHGLRRRCMCLC |
| > non-ACP_120 |
| LPVNEAQCRQVGGYCGLRICNFPSRFLGLCTRNHPCCSRVWV |
| > non-ACP_121 |
| LQDAALGWGRRCPQCPRCPSCPSCPRCPRCPRCKCNPK |
| > non-ACP_122 |
| LRVRLASHLRKLRKRLLRDADDLQKRLAVY |
| > non-ACP_123 |
| LSCKRGTCHFGRCPSHLIKGSCSGG |
| > non-ACP_124 |
| LTCEIDRSLCLLHCRLKGYLRAYCSQQKVCRCVQ |
| > non-ACP_125 |
| MDSNKDERAYAQWVIIILHNVGSSPFKIANLGLSWGKLYADGNKDKEV |
| > non-ACP_126 |
| MHDFWVLWVLLEYIYNSACSVLSATSSVSSRVLNRSLQVKVVKITN |
| > non-ACP_127 |

| |
|---|
| MINRTDCNENSYLEIHNNEGRDTLCFANAGTMPVAIYGVNWVESGNNVVTLQFQRNLSDPRL<br>ETITLQKWGSWNPGHIHEILSIRIY |
| > non-ACP_128 |
| MKTFSVAVAVAIVLAFICTQESSALPVTGVEELVELVSSDDPVADHQELPVELGE<br>RLFNIRKKRASPKCTPYCYPTRDGVFCGVRCDF |
| > non-ACP_129 |
| MKTFSVAVAVAVVLAFICTQESSALPVTGIEELVEPVSSDNNDNHQGLPVELRERLVNIRKKRAP<br>TDCIPYCYPTGDGFHCGVTCRF |
| > non-ACP_130 |
| MLAKIKAMIKKFPNPYTLAAKLTTYEINWYKQQYGRYPWERPVA |
| > non-ACP_131 |
| MNFNKLFVFVALVLAVCIGQSEAGWLKKIGKKIERVGQHTRDATIQTIGVAQ |
| > non-ACP_132 |
| MRILYLLFSVLFLVLQVSPGLSLPQRDMFLCRIGSCHFGRCPIHLVRVGSCFGFRSCCKSPWDV |
| > non-ACP_133 |
| PDEDAINDALNKVCSTGRRQRSICKQLLKK |
| > non-ACP_134 |
| SKRNTWTPSGSNTKWMVEWSGQNLDSGALGTITVDVLRKGN |
| > non-ACP_135 |
| SKYGGECSVEHNTCTYLKGGKDHIVSCPSAANLRCKTERHHCEYDEHHKTVDCQTPV |
| > non-ACP_136 |
| SLQGGAPNFPQPSQQNGGWQVSPDLGRDDKGNTRGQIEIQNKGKDHDFNAGWGKVIRGPNKA<br>KPTWHVGGTYRR |
| > non-ACP_137 |
| SLQPGAPNVNNKDQPWQVSPHISRDDSGNTRTDINVQRHGENNDFEAGWSKVVRGPNKAKP<br>TWHIGGTHRW |
| > non-ACP_138 |
| TYMPVEEGEYIVNISYADQPKKNSPFTAKKQPGPKVDLSGVKAYGPG |
| > non-ACP_139 |
| VLLFLFQAAPGSADAPFADTAACRSQGNFCRAGACPPTFAASGSCHGGLLNCCAK |
| > non-ACP_140 |
| VNYGNGVSCSKTKCSVNWGQAFQERYTAGINSFVSGVASGAGSIGRRP |
| > non-ACP_141 |
| VQETQKLAKTVGANLEETNKKLAPQIKSAYDDFVKQAQEVQKKLHEAASKQ |
| > non-ACP_142 |
| VSCTCRRFSCGFGERASGSCTVNGVRHTLCCRR |
| > non-ACP_143 |
| VTCDILSVEAKGVKLNDAACAAHCLFRGRSGGYCNGKRVCVCR |
| > non-ACP_144 |
| VTCDLLSFEAKGFAANHSLCAAHCLAIGRRGGSCERGVCICRR |
| > non-ACP_145 |
| VTCYCRRTRCGFRERLSGACGYRGRIYRLCCR |
| > non-ACP_146 |
| VTSWSLCTPGCTSPGGGSNCSFCC |
| > non-ACP_147 |

| |
|---|
| WNPFKELERAGQRVRDAIISAGPAVATVAQATALAK |
| > non-ACP_148 |
| YPSDYNGKTVGPDEKIQINSCGRENASSGTEGSFDIVDPNDGNKTIRHFYWECPWG |
| > non-ACP_149 |
| YRQSMNNFQGLRSFGCRFGTCTVQKLAHQIYQFTDKDKDNVAPRSKISPQGY |
| > non-ACP_150 |
| YVPLPNVPQPGRRPFPTFPGQGPFNPKIKWPQGY |

None of the peptides in the independent dataset occurs in Supporting Information S1.

## Supporting Information S3: The predictive results of iACP and Tyagi et al.'s models based on the same independent dataset given in Supporting Information S2

| Peptide ID[a] | Experimental annotations | Predictive results | | iACP |
|---|---|---|---|---|
| | | Tyagi et al.'s method | | |
| | | Module 1 | Module 2 | |
| ACP_1 | Anticancer peptide | Anticancer peptide | Anticancer peptide | Anticancer peptide |
| ACP_2 | Anticancer peptide | Anticancer peptide | Non-Anticancer peptide | Non-Anticancer peptide |
| ACP_3 | Anticancer peptide | Anticancer peptide | Anticancer peptide | Anticancer peptide |
| ACP_4 | Anticancer peptide | Anticancer peptide | Anticancer peptide | Anticancer peptide |
| ACP_5 | Anticancer peptide | Anticancer peptide | Anticancer peptide | Anticancer peptide |
| ACP_6 | Anticancer peptide | Anticancer peptide | Anticancer peptide | Anticancer peptide |
| ACP_7 | Anticancer peptide | Anticancer peptide | Non-Anticancer peptide | Anticancer peptide |
| ACP_8 | Anticancer peptide | Anticancer peptide | Anticancer peptide | Anticancer peptide |
| ACP_9 | Anticancer peptide | Anticancer peptide | Anticancer peptide | Anticancer peptide |
| ACP_10 | Anticancer peptide | Anticancer peptide | Anticancer peptide | Anticancer peptide |
| ACP_11 | Anticancer peptide | Anticancer peptide | Anticancer peptide | Anticancer peptide |
| ACP_12 | Anticancer peptide | Anticancer peptide | Anticancer peptide | Anticancer peptide |
| ACP_13 | Anticancer peptide | Anticancer peptide | Anticancer peptide | Anticancer peptide |
| ACP_14 | Anticancer peptide | Anticancer peptide | Anticancer peptide | Anticancer peptide |
| ACP_15 | Anticancer peptide | Anticancer peptide | Anticancer peptide | Anticancer peptide |
| ACP_16 | Anticancer peptide | Anticancer peptide | Anticancer peptide | Anticancer peptide |
| ACP_17 | Anticancer peptide | Anticancer peptide | Anticancer peptide | Anticancer peptide |
| ACP_18 | Anticancer peptide | Anticancer peptide | Anticancer peptide | Anticancer peptide |
| ACP_19 | Anticancer peptide | Anticancer peptide | Anticancer peptide | Anticancer peptide |
| ACP_20 | Anticancer peptide | Anticancer peptide | Anticancer peptide | Anticancer peptide |
| ACP_21 | Anticancer peptide | Anticancer peptide | Anticancer peptide | Anticancer peptide |
| ACP_22 | Anticancer peptide | Anticancer peptide | Anticancer peptide | Anticancer peptide |
| ACP_23 | Anticancer peptide | Anticancer peptide | Anticancer peptide | Anticancer peptide |
| ACP_24 | Anticancer peptide | Anticancer peptide | Anticancer peptide | Anticancer peptide |
| ACP_25 | Anticancer peptide | Anticancer peptide | Anticancer peptide | Anticancer peptide |
| ACP_26 | Anticancer peptide | Anticancer peptide | Anticancer peptide | Anticancer peptide |
| ACP_27 | Anticancer peptide | Anticancer peptide | Anticancer peptide | Anticancer peptide |
| ACP_28 | Anticancer peptide | Anticancer peptide | Anticancer peptide | Anticancer peptide |
| ACP_29 | Anticancer peptide | Anticancer peptide | Anticancer peptide | Anticancer peptide |
| ACP_30 | Anticancer peptide | Anticancer peptide | Anticancer peptide | Anticancer peptide |

| | | | | |
|---|---|---|---|---|
| ACP_31 | Anticancer peptide | Anticancer peptide | Anticancer peptide | Anticancer peptide |
| ACP_32 | Anticancer peptide | Anticancer peptide | Anticancer peptide | Anticancer peptide |
| ACP_33 | Anticancer peptide | Anticancer peptide | Anticancer peptide | Anticancer peptide |
| ACP_34 | Anticancer peptide | Anticancer peptide | Anticancer peptide | Anticancer peptide |
| ACP_35 | Anticancer peptide | Anticancer peptide | Anticancer peptide | Anticancer peptide |
| ACP_36 | Anticancer peptide | Anticancer peptide | Anticancer peptide | Anticancer peptide |
| ACP_37 | Anticancer peptide | Anticancer peptide | Anticancer peptide | Anticancer peptide |
| ACP_38 | Anticancer peptide | Anticancer peptide | Anticancer peptide | Anticancer peptide |
| ACP_39 | Anticancer peptide | Anticancer peptide | Anticancer peptide | Anticancer peptide |
| ACP_40 | Anticancer peptide | Anticancer peptide | Anticancer peptide | Anticancer peptide |
| ACP_41 | Anticancer peptide | Anticancer peptide | Anticancer peptide | Anticancer peptide |
| ACP_42 | Anticancer peptide | Anticancer peptide | Anticancer peptide | Anticancer peptide |
| ACP_43 | Anticancer peptide | Anticancer peptide | Anticancer peptide | Anticancer peptide |
| ACP_44 | Anticancer peptide | Anticancer peptide | Anticancer peptide | Anticancer peptide |
| ACP_45 | Anticancer peptide | Anticancer peptide | Anticancer peptide | Anticancer peptide |
| ACP_46 | Anticancer peptide | Anticancer peptide | Anticancer peptide | Anticancer peptide |
| ACP_47 | Anticancer peptide | Anticancer peptide | Anticancer peptide | Anticancer peptide |
| ACP_48 | Anticancer peptide | Anticancer peptide | Anticancer peptide | Anticancer peptide |
| ACP_49 | Anticancer peptide | Anticancer peptide | Anticancer peptide | Anticancer peptide |
| ACP_50 | Anticancer peptide | Anticancer peptide | Anticancer peptide | Anticancer peptide |
| ACP_51 | Anticancer peptide | Anticancer peptide | Anticancer peptide | Anticancer peptide |
| ACP_52 | Anticancer peptide | Anticancer peptide | Anticancer peptide | Anticancer peptide |
| ACP_53 | Anticancer peptide | Anticancer peptide | Anticancer peptide | Anticancer peptide |
| ACP_54 | Anticancer peptide | Anticancer peptide | Anticancer peptide | Anticancer peptide |
| ACP_55 | Anticancer peptide | Anticancer peptide | Anticancer peptide | Anticancer peptide |
| ACP_56 | Anticancer peptide | Anticancer peptide | Anticancer peptide | Anticancer peptide |
| ACP_57 | Anticancer peptide | Anticancer peptide | Anticancer peptide | Anticancer peptide |
| ACP_58 | Anticancer peptide | Anticancer peptide | Anticancer peptide | Anticancer peptide |
| ACP_59 | Anticancer peptide | Anticancer peptide | Anticancer peptide | Anticancer peptide |
| ACP_60 | Anticancer peptide | Anticancer peptide | Anticancer peptide | Anticancer peptide |
| ACP_61 | Anticancer peptide | Anticancer peptide | Anticancer peptide | Anticancer peptide |
| ACP_62 | Anticancer peptide | Anticancer peptide | Anticancer peptide | Anticancer peptide |
| ACP_63 | Anticancer peptide | Anticancer peptide | Anticancer peptide | Anticancer peptide |
| ACP_64 | Anticancer peptide | Anticancer peptide | Anticancer peptide | Anticancer peptide |
| ACP_65 | Anticancer peptide | Anticancer peptide | Anticancer peptide | Anticancer peptide |
| ACP_66 | Anticancer peptide | Anticancer peptide | Anticancer peptide | Anticancer peptide |
| ACP_67 | Anticancer peptide | Anticancer peptide | Anticancer peptide | Anticancer peptide |
| ACP_68 | Anticancer peptide | Anticancer peptide | Anticancer peptide | Anticancer peptide |
| ACP_69 | Anticancer peptide | Anticancer peptide | Anticancer peptide | Anticancer peptide |
| ACP_70 | Anticancer peptide | Anticancer peptide | Anticancer peptide | Anticancer peptide |
| ACP_71 | Anticancer peptide | Anticancer peptide | Anticancer peptide | Anticancer peptide |
| ACP_72 | Anticancer peptide | Anticancer peptide | Anticancer peptide | Anticancer peptide |
| ACP_73 | Anticancer peptide | Anticancer peptide | Anticancer peptide | Anticancer peptide |
| ACP_74 | Anticancer peptide | Anticancer peptide | Anticancer peptide | Anticancer peptide |
| ACP_75 | Anticancer peptide | Anticancer peptide | Anticancer peptide | Anticancer peptide |

| | | | | |
|---|---|---|---|---|
| ACP_76 | Anticancer peptide | Anticancer peptide | Anticancer peptide | Anticancer peptide |
| ACP_77 | Anticancer peptide | Anticancer peptide | Anticancer peptide | Anticancer peptide |
| ACP_78 | Anticancer peptide | Anticancer peptide | Anticancer peptide | Anticancer peptide |
| ACP_79 | Anticancer peptide | Anticancer peptide | Anticancer peptide | Anticancer peptide |
| ACP_80 | Anticancer peptide | Anticancer peptide | Anticancer peptide | Anticancer peptide |
| ACP_81 | Anticancer peptide | Anticancer peptide | Anticancer peptide | Anticancer peptide |
| ACP_82 | Anticancer peptide | Anticancer peptide | Anticancer peptide | Anticancer peptide |
| ACP_83 | Anticancer peptide | Anticancer peptide | Anticancer peptide | Anticancer peptide |
| ACP_84 | Anticancer peptide | Anticancer peptide | Anticancer peptide | Anticancer peptide |
| ACP_85 | Anticancer peptide | Anticancer peptide | Anticancer peptide | Anticancer peptide |
| ACP_86 | Anticancer peptide | Anticancer peptide | Anticancer peptide | Anticancer peptide |
| ACP_87 | Anticancer peptide | Anticancer peptide | Anticancer peptide | Anticancer peptide |
| ACP_88 | Anticancer peptide | Anticancer peptide | Anticancer peptide | Anticancer peptide |
| ACP_89 | Anticancer peptide | Anticancer peptide | Anticancer peptide | Anticancer peptide |
| ACP_90 | Anticancer peptide | Anticancer peptide | Anticancer peptide | Anticancer peptide |
| ACP_91 | Anticancer peptide | Anticancer peptide | Anticancer peptide | Anticancer peptide |
| ACP_92 | Anticancer peptide | Anticancer peptide | Anticancer peptide | Anticancer peptide |
| ACP_93 | Anticancer peptide | Anticancer peptide | Anticancer peptide | Anticancer peptide |
| ACP_94 | Anticancer peptide | Anticancer peptide | Non-Anticancer peptide | Non-Anticancer peptide |
| ACP_95 | Anticancer peptide | Anticancer peptide | Anticancer peptide | Anticancer peptide |
| ACP_96 | Anticancer peptide | Anticancer peptide | Non-Anticancer peptide | Non-Anticancer peptide |
| ACP_97 | Anticancer peptide | Anticancer peptide | Anticancer peptide | Anticancer peptide |
| ACP_98 | Anticancer peptide | Anticancer peptide | Anticancer peptide | Anticancer peptide |
| ACP_99 | Anticancer peptide | Anticancer peptide | Anticancer peptide | Anticancer peptide |
| ACP_100 | Anticancer peptide | Anticancer peptide | Non-Anticancer peptide | Non-Anticancer peptide |
| ACP_101 | Anticancer peptide | Anticancer peptide | Non-Anticancer peptide | Non-Anticancer peptide |
| ACP_102 | Anticancer peptide | Anticancer peptide | Anticancer peptide | Non-Anticancer peptide |
| ACP_103 | Anticancer peptide | Anticancer peptide | Anticancer peptide | Anticancer peptide |
| ACP_104 | Anticancer peptide | Anticancer peptide | Anticancer peptide | Non-Anticancer peptide |
| ACP_105 | Anticancer peptide | Anticancer peptide | Anticancer peptide | Anticancer peptide |
| ACP_106 | Anticancer peptide | Anticancer peptide | Anticancer peptide | Anticancer peptide |
| ACP_107 | Anticancer peptide | Anticancer peptide | Anticancer peptide | Anticancer peptide |
| ACP_108 | Anticancer peptide | Anticancer peptide | Non-Anticancer peptide | Anticancer peptide |
| ACP_109 | Anticancer peptide | Anticancer peptide | Anticancer peptide | Anticancer peptide |
| ACP_110 | Anticancer peptide | Anticancer peptide | Anticancer peptide | Anticancer peptide |
| ACP_111 | Anticancer peptide | Anticancer peptide | Anticancer peptide | Anticancer peptide |
| ACP_112 | Anticancer peptide | Anticancer peptide | Anticancer peptide | Anticancer peptide |
| ACP_113 | Anticancer peptide | Anticancer peptide | Anticancer peptide | Anticancer peptide |
| ACP_114 | Anticancer peptide | Anticancer peptide | Anticancer peptide | Anticancer peptide |
| ACP_115 | Anticancer peptide | Anticancer peptide | Anticancer peptide | Anticancer peptide |
| ACP_116 | Anticancer peptide | Anticancer peptide | Anticancer peptide | Anticancer peptide |
| ACP_117 | Anticancer peptide | Anticancer peptide | Anticancer peptide | Anticancer peptide |
| ACP_118 | Anticancer peptide | Anticancer peptide | Anticancer peptide | Anticancer peptide |
| ACP_119 | Anticancer peptide | Anticancer peptide | Anticancer peptide | Anticancer peptide |
| ACP_120 | Anticancer peptide | Anticancer peptide | Anticancer peptide | Anticancer peptide |

| | | | | |
|---|---|---|---|---|
| ACP_121 | Anticancer peptide | Anticancer peptide | Anticancer peptide | Anticancer peptide |
| ACP_122 | Anticancer peptide | Anticancer peptide | Anticancer peptide | Anticancer peptide |
| ACP_123 | Anticancer peptide | Anticancer peptide | Anticancer peptide | Anticancer peptide |
| ACP_124 | Anticancer peptide | Anticancer peptide | Anticancer peptide | Anticancer peptide |
| ACP_125 | Anticancer peptide | Anticancer peptide | Anticancer peptide | Anticancer peptide |
| ACP_126 | Anticancer peptide | Anticancer peptide | Anticancer peptide | Anticancer peptide |
| ACP_127 | Anticancer peptide | Anticancer peptide | Anticancer peptide | Anticancer peptide |
| ACP_128 | Anticancer peptide | Anticancer peptide | Anticancer peptide | Anticancer peptide |
| ACP_129 | Anticancer peptide | Anticancer peptide | Anticancer peptide | Anticancer peptide |
| ACP_130 | Anticancer peptide | Anticancer peptide | Anticancer peptide | Anticancer peptide |
| ACP_131 | Anticancer peptide | Anticancer peptide | Anticancer peptide | Anticancer peptide |
| ACP_132 | Anticancer peptide | Anticancer peptide | Anticancer peptide | Anticancer peptide |
| ACP_133 | Anticancer peptide | Anticancer peptide | Anticancer peptide | Anticancer peptide |
| ACP_134 | Anticancer peptide | Anticancer peptide | Anticancer peptide | Anticancer peptide |
| ACP_135 | Anticancer peptide | Anticancer peptide | Non-Anticancer peptide | Anticancer peptide |
| ACP_136 | Anticancer peptide | Anticancer peptide | Non-Anticancer peptide | Non-Anticancer peptide |
| ACP_137 | Anticancer peptide | Anticancer peptide | Non-Anticancer peptide | Anticancer peptide |
| ACP_138 | Anticancer peptide | Anticancer peptide | Anticancer peptide | Anticancer peptide |
| ACP_139 | Anticancer peptide | Anticancer peptide | Non-Anticancer peptide | Anticancer peptide |
| ACP_140 | Anticancer peptide | Anticancer peptide | Non-Anticancer peptide | Anticancer peptide |
| ACP_141 | Anticancer peptide | Anticancer peptide | Anticancer peptide | Anticancer peptide |
| ACP_142 | Anticancer peptide | Anticancer peptide | Non-Anticancer peptide | Anticancer peptide |
| ACP_143 | Anticancer peptide | Anticancer peptide | Anticancer peptide | Anticancer peptide |
| ACP_144 | Anticancer peptide | Anticancer peptide | Non-Anticancer peptide | Non-Anticancer peptide |
| ACP_145 | Anticancer peptide | Anticancer peptide | Non-Anticancer peptide | Non-Anticancer peptide |
| ACP_146 | Anticancer peptide | Anticancer peptide | Anticancer peptide | Anticancer peptide |
| ACP_147 | Anticancer peptide | Anticancer peptide | Non-Anticancer peptide | Anticancer peptide |
| ACP_148 | Anticancer peptide | Anticancer peptide | Anticancer peptide | Anticancer peptide |
| ACP_149 | Anticancer peptide | Anticancer peptide | Anticancer peptide | Anticancer peptide |
| ACP_150 | Anticancer peptide | Anticancer peptide | Anticancer peptide | Anticancer peptide |
| non-ACP_1 | non-Anticancer peptide | Anticancer peptide | Non-Anticancer peptide | Non-Anticancer peptide |
| non-ACP_2 | non-Anticancer peptide | Anticancer peptide | Anticancer peptide | Non-Anticancer peptide |
| non-ACP_3 | non-Anticancer peptide | Anticancer peptide | Anticancer peptide | Non-Anticancer peptide |
| non-ACP_4 | non-Anticancer peptide | Anticancer peptide | Anticancer peptide | Non-Anticancer peptide |
| non-ACP_5 | non-Anticancer peptide | Anticancer peptide | Anticancer peptide | Non-Anticancer peptide |
| non-ACP_6 | non-Anticancer peptide | Anticancer peptide | Anticancer peptide | Non-Anticancer peptide |
| non-ACP_7 | non-Anticancer peptide | Anticancer peptide | Non-Anticancer peptide | Non-Anticancer peptide |
| non-ACP_8 | non-Anticancer peptide | Anticancer peptide | Non-Anticancer peptide | Non-Anticancer peptide |
| non-ACP_9 | non-Anticancer peptide | Anticancer peptide | Non-Anticancer peptide | Non-Anticancer peptide |
| non-ACP_10 | non-Anticancer peptide | Anticancer peptide | Anticancer peptide | Non-Anticancer peptide |
| non-ACP_11 | non-Anticancer peptide | Anticancer peptide | Non-Anticancer peptide | Non-Anticancer peptide |
| non-ACP_12 | non-Anticancer peptide | Anticancer peptide | Non-Anticancer peptide | Non-Anticancer peptide |
| non-ACP_13 | non-Anticancer peptide | Anticancer peptide | Anticancer peptide | Non-Anticancer peptide |
| non-ACP_14 | non-Anticancer peptide | Anticancer peptide | Anticancer peptide | Non-Anticancer peptide |
| non-ACP_15 | non-Anticancer peptide | Anticancer peptide | Non-Anticancer peptide | Non-Anticancer peptide |

| | | | | |
|---|---|---|---|---|
| non-ACP_16 | non-Anticancer peptide | Anticancer peptide | Anticancer peptide | Non-Anticancer peptide |
| non-ACP_17 | non-Anticancer peptide | Anticancer peptide | Anticancer peptide | Non-Anticancer peptide |
| non-ACP_18 | non-Anticancer peptide | Anticancer peptide | Anticancer peptide | Non-Anticancer peptide |
| non-ACP_19 | non-Anticancer peptide | Anticancer peptide | Anticancer peptide | Non-Anticancer peptide |
| non-ACP_20 | non-Anticancer peptide | Anticancer peptide | Anticancer peptide | Non-Anticancer peptide |
| non-ACP_21 | non-Anticancer peptide | Anticancer peptide | Non-Anticancer peptide | Non-Anticancer peptide |
| non-ACP_22 | non-Anticancer peptide | Anticancer peptide | Non-Anticancer peptide | Non-Anticancer peptide |
| non-ACP_23 | non-Anticancer peptide | Anticancer peptide | Anticancer peptide | Non-Anticancer peptide |
| non-ACP_24 | non-Anticancer peptide | Anticancer peptide | Non-Anticancer peptide | Non-Anticancer peptide |
| non-ACP_25 | non-Anticancer peptide | Anticancer peptide | Anticancer peptide | Non-Anticancer peptide |
| non-ACP_26 | non-Anticancer peptide | Anticancer peptide | Anticancer peptide | Non-Anticancer peptide |
| non-ACP_27 | non-Anticancer peptide | Anticancer peptide | Anticancer peptide | Non-Anticancer peptide |
| non-ACP_28 | non-Anticancer peptide | Anticancer peptide | Non-Anticancer peptide | Non-Anticancer peptide |
| non-ACP_29 | non-Anticancer peptide | Anticancer peptide | Non-Anticancer peptide | Non-Anticancer peptide |
| non-ACP_30 | non-Anticancer peptide | Anticancer peptide | Anticancer peptide | Non-Anticancer peptide |
| non-ACP_31 | non-Anticancer peptide | Anticancer peptide | Non-Anticancer peptide | Non-Anticancer peptide |
| non-ACP_32 | non-Anticancer peptide | Anticancer peptide | Non-Anticancer peptide | Non-Anticancer peptide |
| non-ACP_33 | non-Anticancer peptide | Anticancer peptide | Non-Anticancer peptide | Non-Anticancer peptide |
| non-ACP_34 | non-Anticancer peptide | Anticancer peptide | Non-Anticancer peptide | Non-Anticancer peptide |
| non-ACP_35 | non-Anticancer peptide | Anticancer peptide | Non-Anticancer peptide | Non-Anticancer peptide |
| non-ACP_36 | non-Anticancer peptide | Anticancer peptide | Anticancer peptide | Non-Anticancer peptide |
| non-ACP_37 | non-Anticancer peptide | Anticancer peptide | Non-Anticancer peptide | Non-Anticancer peptide |
| non-ACP_38 | non-Anticancer peptide | Anticancer peptide | Non-Anticancer peptide | Non-Anticancer peptide |
| non-ACP_39 | non-Anticancer peptide | Anticancer peptide | Anticancer peptide | Non-Anticancer peptide |
| non-ACP_40 | non-Anticancer peptide | Anticancer peptide | Non-Anticancer peptide | Non-Anticancer peptide |
| non-ACP_41 | non-Anticancer peptide | Anticancer peptide | Non-Anticancer peptide | Non-Anticancer peptide |
| non-ACP_42 | non-Anticancer peptide | Anticancer peptide | Non-Anticancer peptide | Non-Anticancer peptide |
| non-ACP_43 | non-Anticancer peptide | Anticancer peptide | Anticancer peptide | Non-Anticancer peptide |
| non-ACP_44 | non-Anticancer peptide | Anticancer peptide | Non-Anticancer peptide | Non-Anticancer peptide |
| non-ACP_45 | non-Anticancer peptide | Anticancer peptide | Non-Anticancer peptide | Non-Anticancer peptide |
| non-ACP_46 | non-Anticancer peptide | Anticancer peptide | Non-Anticancer peptide | Non-Anticancer peptide |
| non-ACP_47 | non-Anticancer peptide | Anticancer peptide | Anticancer peptide | Non-Anticancer peptide |
| non-ACP_48 | non-Anticancer peptide | Anticancer peptide | Anticancer peptide | Non-Anticancer peptide |
| non-ACP_49 | non-Anticancer peptide | Anticancer peptide | Non-Anticancer peptide | Non-Anticancer peptide |
| non-ACP_50 | non-Anticancer peptide | Anticancer peptide | Non-Anticancer peptide | Non-Anticancer peptide |
| non-ACP_51 | non-Anticancer peptide | Anticancer peptide | Anticancer peptide | Non-Anticancer peptide |
| non-ACP_52 | non-Anticancer peptide | Anticancer peptide | Anticancer peptide | Non-Anticancer peptide |
| non-ACP_53 | non-Anticancer peptide | Anticancer peptide | Anticancer peptide | Non-Anticancer peptide |
| non-ACP_54 | non-Anticancer peptide | Anticancer peptide | Anticancer peptide | Non-Anticancer peptide |
| non-ACP_55 | non-Anticancer peptide | Anticancer peptide | Anticancer peptide | Non-Anticancer peptide |
| non-ACP_56 | non-Anticancer peptide | Anticancer peptide | Anticancer peptide | Non-Anticancer peptide |
| non-ACP_57 | non-Anticancer peptide | Anticancer peptide | Anticancer peptide | Non-Anticancer peptide |
| non-ACP_58 | non-Anticancer peptide | Anticancer peptide | Anticancer peptide | Non-Anticancer peptide |
| non-ACP_59 | non-Anticancer peptide | Anticancer peptide | Anticancer peptide | Non-Anticancer peptide |
| non-ACP_60 | non-Anticancer peptide | Anticancer peptide | Anticancer peptide | Non-Anticancer peptide |

| | | | | |
|---|---|---|---|---|
| non-ACP_61 | non-Anticancer peptide | Anticancer peptide | Non-Anticancer peptide | Non-Anticancer peptide |
| non-ACP_62 | non-Anticancer peptide | Anticancer peptide | Non-Anticancer peptide | Non-Anticancer peptide |
| non-ACP_63 | non-Anticancer peptide | Anticancer peptide | Anticancer peptide | Non-Anticancer peptide |
| non-ACP_64 | non-Anticancer peptide | Anticancer peptide | Anticancer peptide | Non-Anticancer peptide |
| non-ACP_65 | non-Anticancer peptide | Anticancer peptide | Non-Anticancer peptide | Non-Anticancer peptide |
| non-ACP_66 | non-Anticancer peptide | Anticancer peptide | Anticancer peptide | Non-Anticancer peptide |
| non-ACP_67 | non-Anticancer peptide | Anticancer peptide | Anticancer peptide | Non-Anticancer peptide |
| non-ACP_68 | non-Anticancer peptide | Anticancer peptide | Anticancer peptide | Non-Anticancer peptide |
| non-ACP_69 | non-Anticancer peptide | Anticancer peptide | Anticancer peptide | Non-Anticancer peptide |
| non-ACP_70 | non-Anticancer peptide | Anticancer peptide | Anticancer peptide | Non-Anticancer peptide |
| non-ACP_71 | non-Anticancer peptide | Anticancer peptide | Anticancer peptide | Non-Anticancer peptide |
| non-ACP_72 | non-Anticancer peptide | Anticancer peptide | Non-Anticancer peptide | Non-Anticancer peptide |
| non-ACP_73 | non-Anticancer peptide | Anticancer peptide | Anticancer peptide | Non-Anticancer peptide |
| non-ACP_74 | non-Anticancer peptide | Anticancer peptide | Anticancer peptide | Non-Anticancer peptide |
| non-ACP_75 | non-Anticancer peptide | Anticancer peptide | Anticancer peptide | Non-Anticancer peptide |
| non-ACP_76 | non-Anticancer peptide | Anticancer peptide | Anticancer peptide | Non-Anticancer peptide |
| non-ACP_77 | non-Anticancer peptide | Anticancer peptide | Anticancer peptide | Non-Anticancer peptide |
| non-ACP_78 | non-Anticancer peptide | Anticancer peptide | Anticancer peptide | Non-Anticancer peptide |
| non-ACP_79 | non-Anticancer peptide | Anticancer peptide | Anticancer peptide | Non-Anticancer peptide |
| non-ACP_80 | non-Anticancer peptide | Anticancer peptide | Non-Anticancer peptide | Non-Anticancer peptide |
| non-ACP_81 | non-Anticancer peptide | Anticancer peptide | Anticancer peptide | Non-Anticancer peptide |
| non-ACP_82 | non-Anticancer peptide | Anticancer peptide | Anticancer peptide | Non-Anticancer peptide |
| non-ACP_83 | non-Anticancer peptide | Anticancer peptide | Anticancer peptide | Anticancer peptide |
| non-ACP_84 | non-Anticancer peptide | Anticancer peptide | Non-Anticancer peptide | Anticancer peptide |
| non-ACP_85 | non-Anticancer peptide | Anticancer peptide | Anticancer peptide | Anticancer peptide |
| non-ACP_86 | non-Anticancer peptide | Anticancer peptide | Anticancer peptide | Anticancer peptide |
| non-ACP_87 | non-Anticancer peptide | Anticancer peptide | Non-Anticancer peptide | Non-Anticancer peptide |
| non-ACP_88 | non-Anticancer peptide | Anticancer peptide | Non-Anticancer peptide | Anticancer peptide |
| non-ACP_89 | non-Anticancer peptide | Anticancer peptide | Anticancer peptide | Anticancer peptide |
| non-ACP_90 | non-Anticancer peptide | Anticancer peptide | Anticancer peptide | Anticancer peptide |
| non-ACP_91 | non-Anticancer peptide | Anticancer peptide | Anticancer peptide | Non-Anticancer peptide |
| non-ACP_92 | non-Anticancer peptide | Anticancer peptide | Anticancer peptide | Non-Anticancer peptide |
| non-ACP_93 | non-Anticancer peptide | Anticancer peptide | Anticancer peptide | Non-Anticancer peptide |
| non-ACP_94 | non-Anticancer peptide | Anticancer peptide | Anticancer peptide | Non-Anticancer peptide |
| non-ACP_95 | non-Anticancer peptide | Anticancer peptide | Anticancer peptide | Non-Anticancer peptide |
| non-ACP_96 | non-Anticancer peptide | Anticancer peptide | Anticancer peptide | Non-Anticancer peptide |
| non-ACP_97 | non-Anticancer peptide | Anticancer peptide | Non-Anticancer peptide | Non-Anticancer peptide |
| non-ACP_98 | non-Anticancer peptide | Anticancer peptide | Anticancer peptide | Non-Anticancer peptide |
| non-ACP_99 | non-Anticancer peptide | Anticancer peptide | Non-Anticancer peptide | Non-Anticancer peptide |
| non-ACP_100 | non-Anticancer peptide | Anticancer peptide | Non-Anticancer peptide | Non-Anticancer peptide |
| non-ACP_101 | non-Anticancer peptide | Anticancer peptide | Anticancer peptide | Non-Anticancer peptide |
| non-ACP_102 | non-Anticancer peptide | Anticancer peptide | Non-Anticancer peptide | Non-Anticancer peptide |
| non-ACP_103 | non-Anticancer peptide | Anticancer peptide | Anticancer peptide | Non-Anticancer peptide |
| non-ACP_104 | non-Anticancer peptide | Anticancer peptide | Anticancer peptide | Non-Anticancer peptide |
| non-ACP_105 | non-Anticancer peptide | Anticancer peptide | Non-Anticancer peptide | Non-Anticancer peptide |

| | | | | |
|---|---|---|---|---|
| non-ACP_106 | non-Anticancer peptide | Anticancer peptide | Non-Anticancer peptide | Non-Anticancer peptide |
| non-ACP_107 | non-Anticancer peptide | Anticancer peptide | Non-Anticancer peptide | Non-Anticancer peptide |
| non-ACP_108 | non-Anticancer peptide | Anticancer peptide | Anticancer peptide | Non-Anticancer peptide |
| non-ACP_109 | non-Anticancer peptide | Anticancer peptide | Non-Anticancer peptide | Non-Anticancer peptide |
| non-ACP_110 | non-Anticancer peptide | Anticancer peptide | Anticancer peptide | Non-Anticancer peptide |
| non-ACP_111 | non-Anticancer peptide | Anticancer peptide | Non-Anticancer peptide | Non-Anticancer peptide |
| non-ACP_112 | non-Anticancer peptide | Anticancer peptide | Anticancer peptide | Non-Anticancer peptide |
| non-ACP_113 | non-Anticancer peptide | Anticancer peptide | Non-Anticancer peptide | Non-Anticancer peptide |
| non-ACP_114 | non-Anticancer peptide | Anticancer peptide | Non-Anticancer peptide | Non-Anticancer peptide |
| non-ACP_115 | non-Anticancer peptide | Anticancer peptide | Anticancer peptide | Non-Anticancer peptide |
| non-ACP_116 | non-Anticancer peptide | Anticancer peptide | Anticancer peptide | Non-Anticancer peptide |
| non-ACP_117 | non-Anticancer peptide | Anticancer peptide | Non-Anticancer peptide | Non-Anticancer peptide |
| non-ACP_118 | non-Anticancer peptide | Anticancer peptide | Anticancer peptide | Non-Anticancer peptide |
| non-ACP_119 | non-Anticancer peptide | Anticancer peptide | Anticancer peptide | Non-Anticancer peptide |
| non-ACP_120 | non-Anticancer peptide | Anticancer peptide | Anticancer peptide | Non-Anticancer peptide |
| non-ACP_121 | non-Anticancer peptide | Anticancer peptide | Anticancer peptide | Non-Anticancer peptide |
| non-ACP_122 | non-Anticancer peptide | Anticancer peptide | Non-Anticancer peptide | Non-Anticancer peptide |
| non-ACP_123 | non-Anticancer peptide | Anticancer peptide | Anticancer peptide | Non-Anticancer peptide |
| non-ACP_124 | non-Anticancer peptide | Anticancer peptide | Anticancer peptide | Non-Anticancer peptide |
| non-ACP_125 | non-Anticancer peptide | Anticancer peptide | Non-Anticancer peptide | Non-Anticancer peptide |
| non-ACP_126 | non-Anticancer peptide | Anticancer peptide | Non-Anticancer peptide | Non-Anticancer peptide |
| non-ACP_127 | non-Anticancer peptide | Anticancer peptide | Non-Anticancer peptide | Non-Anticancer peptide |
| non-ACP_128 | non-Anticancer peptide | Anticancer peptide | Non-Anticancer peptide | Non-Anticancer peptide |
| non-ACP_129 | non-Anticancer peptide | Anticancer peptide | Non-Anticancer peptide | Non-Anticancer peptide |
| non-ACP_130 | non-Anticancer peptide | Anticancer peptide | Non-Anticancer peptide | Non-Anticancer peptide |
| non-ACP_131 | non-Anticancer peptide | Anticancer peptide | Non-Anticancer peptide | Anticancer peptide |
| non-ACP_132 | non-Anticancer peptide | Anticancer peptide | Anticancer peptide | Non-Anticancer peptide |
| non-ACP_133 | non-Anticancer peptide | Anticancer peptide | Non-Anticancer peptide | Non-Anticancer peptide |
| non-ACP_134 | non-Anticancer peptide | Anticancer peptide | Non-Anticancer peptide | Non-Anticancer peptide |
| non-ACP_135 | non-Anticancer peptide | Anticancer peptide | Non-Anticancer peptide | Non-Anticancer peptide |
| non-ACP_136 | non-Anticancer peptide | Anticancer peptide | Non-Anticancer peptide | Non-Anticancer peptide |
| non-ACP_137 | non-Anticancer peptide | Anticancer peptide | Non-Anticancer peptide | Non-Anticancer peptide |
| non-ACP_138 | non-Anticancer peptide | Anticancer peptide | Non-Anticancer peptide | Non-Anticancer peptide |
| non-ACP_139 | non-Anticancer peptide | Anticancer peptide | Anticancer peptide | Non-Anticancer peptide |
| non-ACP_140 | non-Anticancer peptide | Anticancer peptide | Anticancer peptide | Anticancer peptide |
| non-ACP_141 | non-Anticancer peptide | Anticancer peptide | Non-Anticancer peptide | Anticancer peptide |
| non-ACP_142 | non-Anticancer peptide | Anticancer peptide | Anticancer peptide | Non-Anticancer peptide |
| non-ACP_143 | non-Anticancer peptide | Anticancer peptide | Anticancer peptide | Anticancer peptide |
| non-ACP_144 | non-Anticancer peptide | Anticancer peptide | Anticancer peptide | Anticancer peptide |
| non-ACP_145 | non-Anticancer peptide | Anticancer peptide | Anticancer peptide | Non-Anticancer peptide |
| non-ACP_146 | non-Anticancer peptide | Anticancer peptide | Anticancer peptide | Non-Anticancer peptide |
| non-ACP_147 | non-Anticancer peptide | Anticancer peptide | Non-Anticancer peptide | Non-Anticancer peptide |
| non-ACP_148 | non-Anticancer peptide | Anticancer peptide | Non-Anticancer peptide | Non-Anticancer peptide |
| non-ACP_149 | non-Anticancer peptide | Anticancer peptide | Non-Anticancer peptide | Non-Anticancer peptide |
| non-ACP_150 | non-Anticancer peptide | Anticancer peptide | Anticancer peptide | Non-Anticancer peptide |

[a]Peptide ID is in accordance with the sequence ID in the independent dataset provided in Supporting Information S2.

**Supporting Information S4: List of the 126 optimal one-gap dipeptides and their *F-scores***

| Dipeptide | F-score | Dipeptide | *F-score* | Dipeptide | *F-score* | Dipeptide | *F-score* | Dipeptide | *F-score* |
|---|---|---|---|---|---|---|---|---|---|
| AK | 38.03 | CN | 16.30 | LP | 8.93 | EL | 6.19 | NV | 4.61 |
| KC | 34.43 | AR | 15.79 | VL | 8.59 | EQ | 6.19 | YP | 4.47 |
| EC | 34.14 | CS | 15.32 | VG | 8.56 | PK | 6.10 | MI | 4.45 |
| CE | 34.02 | FI | 14.96 | EP | 8.31 | PN | 6.08 | HV | 4.43 |
| IK | 31.22 | GR | 14.79 | QE | 7.93 | LN | 6.07 | IY | 4.41 |
| KF | 30.09 | CK | 14.75 | VI | 7.89 | DM | 6.06 | VN | 4.41 |
| PI | 28.37 | PP | 14.04 | AL | 7.84 | RQ | 5.91 | FV | 4.30 |
| KI | 28.01 | GS | 13.78 | LE | 7.80 | NS | 5.83 | YC | 4.28 |
| LL | 27.60 | YN | 13.75 | QP | 7.62 | CI | 5.81 | NR | 4.22 |
| VY | 27.37 | AA | 13.65 | RT | 7.58 | EN | 5.78 | WQ | 4.17 |
| IC | 27.16 | CW | 13.00 | AQ | 7.58 | SC | 5.62 | RS | 4.14 |
| CC | 26.86 | QQ | 12.99 | ES | 7.52 | NL | 5.48 | MV | 4.11 |
| SK | 25.78 | CT | 12.83 | IA | 7.49 | ND | 5.45 | CL | 4.08 |
| KK | 25.58 | LR | 12.80 | LA | 7.42 | SI | 5.43 | LF | 4.03 |
| FK | 22.53 | QS | 12.66 | PC | 7.41 | WP | 5.43 | QT | 4.01 |
| IG | 20.92 | IS | 12.56 | PQ | 7.15 | EA | 5.42 | EE | 4.01 |
| GF | 20.88 | KV | 12.50 | AE | 7.01 | VM | 5.29 | GV | 3.90 |
| GI | 20.75 | QR | 11.10 | LV | 6.98 | QK | 5.15 | RM | 3.88 |
| KA | 19.65 | SY | 10.61 | MA | 6.97 | PD | 4.92 | | |
| VK | 19.55 | FG | 10.36 | DQ | 6.95 | GL | 4.89 | | |
| SV | 19.37 | TQ | 10.01 | AM | 6.88 | QV | 4.83 | | |
| AP | 18.56 | RR | 9.98 | CR | 6.82 | SR | 4.83 | | |
| LK | 18.10 | CF | 9.86 | AH | 6.70 | ER | 4.83 | | |
| KL | 18.04 | FP | 9.70 | SQ | 6.46 | TE | 4.81 | | |
| RA | 17.34 | WK | 9.42 | PT | 6.43 | QL | 4.76 | | |
| RL | 17.17 | FC | 9.15 | TP | 6.39 | GD | 4.74 | | |
| LQ | 17.15 | PA | 9.07 | RD | 6.29 | DD | 4.70 | | |
